# Investigating coordinated account creation using burst detection and network analysis

**DOI:** 10.1186/s40537-023-00695-7

**Published:** 2023-02-10

**Authors:** Daniele Bellutta, Kathleen M. Carley

**Affiliations:** CASOS & IDeaS Centers, Carnegie Mellon University, Pittsburgh, PA USA

**Keywords:** Misinformation, Elections, Social networks

## Abstract

Democracies around the world face the threat of manipulation of their electorates via coordinated online influence campaigns. Researchers have responded by developing valuable methods for finding automated accounts and identifying false information, but these valiant efforts often fall into a cat-and-mouse game with perpetrators who constantly change their behavior. This has forced several researchers to go beyond the detection of individual malicious actors by instead identifying the coordinated activity that propels potent information operations. In this vein, we provide rigorous quantitative evidence for the notion that sudden increases in Twitter account creations may provide early warnings of online information operations. Analysis of fourteen months of tweets discussing the 2020 U.S. elections revealed that accounts created during bursts exhibited more similar behavior, showed more agreement on mail-in voting and mask wearing, and were more likely to be bots and share links to low-credibility sites. In concert with other techniques for detecting nefarious activity, social media platforms could temporarily limit the influence of accounts created during these bursts. Given the advantages of combining multiple anti-misinformation methods, we join others in presenting a case for the need to develop more integrable methods for countering online influence campaigns.

**Supplementary Information:**

The online version contains supplementary material available at 10.1186/s40537-023-00695-7.

## Introduction

Worldwide concern about the orchestrated manipulation of social media has only grown since the discovery of online influence campaigns carried out during the 2016 U.S. presidential election [1]. This global threat to democracy [2] led to the emergence of the field of social cybersecurity [3, 4] and cast a dark cloud over the following U.S. presidential election in 2020.

**Fig. 1:**
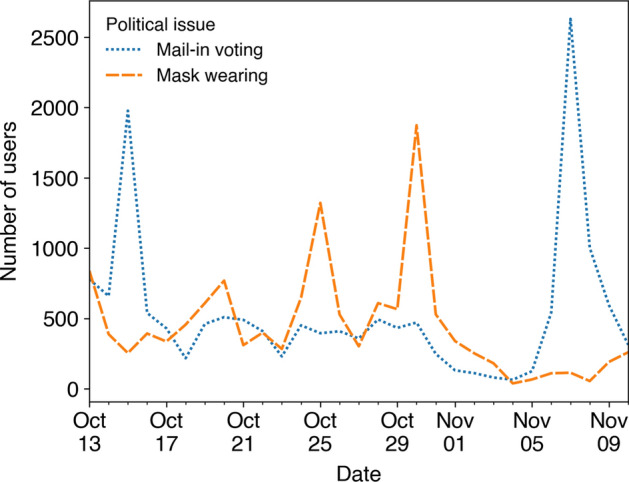
The daily number of users mentioning partisan hashtags on mail-in voting or mask wearing in late 2020. These hashtags are listed in Additional file 2: Methods

Instituted in response to the COVID-19 pandemic, expansions of mail-in voting and mask wearing regulations became extremely controversial during the 2020 U.S. elections [5, 6]. In fact, Fig. 1 reveals a decrease in sectarian discussion of these topics on Twitter around the time of the presidential election, with partisan discussion of mail-in voting then spiking on the day the result of the election was called [7]. The controversy surrounding these two issues made them particularly vulnerable targets for coordinated nefarious activity, which may have contributed to these changes in their discussion on Twitter.

What has made influence campaigns particularly threatening has indeed been the coordination employed in spreading disinformation, with some state actors making use of vast government resources to attack rivals [8, 9]. Yet, the issue of discovering coordinated activity has received relatively little attention [10, 11] compared to the identification of false narratives [12, 13] and automated accounts [14–16]. Though work to detect individual malicious accounts and messages is incredibly valuable and necessary for slowing the spread of misinformation, such efforts often fall into a cat-and-mouse game [10, 11, 17] with perpetrators who constantly change their behavior, even forcing the adoption of adversarial methods for some tasks [18]. This increasing sophistication makes the detection of individual inauthentic actors more difficult, but these actors’ basic goal of gaining influence by manipulating social media platforms’ recommendation algorithms remains the same [10]. Given that accomplishing this goal requires the coordinated use of multiple accounts, several researchers have recognized the need to complement the detection of individual malicious accounts and messages with the identification of the coordinated activity that propels potent information operations [10, 11, 19].

Recent work has also highlighted the importance of intervening early to counter the spread of misinformation. Nudging people to consider accuracy before posting online can reduce misinformation sharing [20], and platform policies of removing misleading content and accounts can significantly reduce the influence of anti-vaccine communities [21]. Responding early is particularly important given that later interventions can decrease belief in true information [22] and that organizations struggle to broadcast their corrections of misinformation [23].

We therefore sought to investigate influence campaigns from the moment of their birth, before their activities reach levels noticeable by more archetypal coordination detectors. Our approach began by aggregating the creation dates of accounts featured in more than 541 million tweets discussing American politics between December 2019 and February 2021. Though a larger Twitter data set on the 2020 elections is known to exist [24], published works using those data have thus far only analyzed smaller subsets of that collection [25–27]. To our knowledge, this makes our data the largest fully analyzed set of tweets on the 2020 U.S. elections to date.

Using this massive data set, we discovered that certain days showed abnormal spikes in the number of accounts created that later tweeted about the 2020 elections. We then found that when compared to accounts created during non-peak times, those created during bursts

1. exhibited more similar behavior amongst themselves,
2. agreed more on mail-in voting and mask wearing,
3. were more likely to be bots, and
4. were more likely to share links to low-credibility sources.

Given that abnormally rapid account creation may warn of malicious coordination, social media platforms could pre-emptively reduce the influence of these suspicious accounts. Though this technique cannot be used in isolation because of the genuine users who would be caught in the crossfire, this strategy could be used as part of a pipeline of interoperable tools [28] that together gradually build evidence of maleficence against suspicious accounts, allowing online platforms to incrementally sanction actors as additional evidence makes their harmfulness more clear. Since social media companies have already implemented similar systems to derank messages and accounts based on other criteria [29, 30], we believe researchers should prioritize the development of interoperable methods that, though perhaps insufficient on their own, can be combined to produce more robust and versatile systems for countering online information operations.

## Related work

### Coordination detection

Researchers studying social media coordination have made a few notable developments on frameworks for detecting malicious coordination. One of the earlier works in this area, DeBot, identified Twitter bots by finding accounts with similar timing for their activity [31]. Later, a different study identified malicious accounts individually but then connected them together based on similar activity in order to determine which accounts were most influential [19].

Recent work has focused on detecting coordination via frameworks that connect accounts on the basis of similar behavior. One study was able to predict certain future activities of information operations by first forming user networks based on behavioral similarities like shared hashtag use and then generating input features for a random forest classifier [32]. Pacheco et al. instead developed a general framework in which accounts are connected together with edges weighted based on some kind of similarity measure, such as the similarity of their hashtag use or image sharing [11]. The authors then clustered the network to find groups of accounts that may have been coordinating their actions [11]. Magelinski et al. then extended this framework to use multiple kinds of behavioral similarity at the same time by creating multi-level networks between accounts, with each level using a different similarity measure [33]. Their framework then used multi-view modularity clustering to cluster the entire multi-level network [33]. Pacheco et al. and Magelinski et al. also briefly mentioned using account creation within their frameworks to detect coordination, but they did not investigate this possibility.

### Account creation bursts

Though account age has been used to study the nature of Twitter users in several instances [34–37], only a few works have analyzed the aggregated creation times of multiple accounts. In an early study of this matter, Lee and Kim studied hour-long time periods in which most of the created accounts were later suspended [38]. Though they found that such periods only constituted a small percentage of all creation times, the rest of their analysis instead focused on clustering the screen names of accounts created together to find suspicious groups of accounts [38]. Lee and Kim further noted that their strategy could not be used to create a full coordination detection system, because it could not conclusively determine whether an account is malicious without considering more information [38]. Our own work studying account creation bursts features the same restriction, meaning it must be combined with other coordination detection techniques.

Some analyst reports of social media influence campaigns have also mentioned groups of accounts created together for manipulative purposes. In 2019, Twitter reported it had suspended thousands of accounts that were created together after it had already suspended a set of accounts belonging to the People’s Republic of China [39]. According to the company, these accounts were pre-emptively suspended before they were “substantially active” [39]. In 2020, a Graphika report mentioned the creation over a few months of tens of inauthentic accounts created in bursts that each lasted a few days [40]. The following year, Facebook reported on nearly one hundred recently created Instagram accounts used by Russia’s Internet Research Agency [41]. The company further detailed that its automated systems block millions of fake accounts per day, “often minutes after creation” [41, p. 35]. This demonstrates that social media platforms have already developed early detection technologies for identifying inauthentic accounts but does not specify how they function.

Meanwhile, only a few studies have sought to systemically analyze larger bursts of account creations. Takacs and McCulloh discovered bursts of created accounts that followed candidates in the 2018 U.S. Senate elections [42]. They found that the accounts created during and after these bursts exhibited statistically different relationships between their numbers of friends, followers, and posts when compared to the candidates’ followers that were created before these spikes [42]. Noting that many of these suspicious accounts displayed no tweeting activity, the authors also stated that they could be dormant bots [42]. However, the authors did not study these accounts’ behaviors, nor did they examine bursts of account creations in the broader Twitter user base. In a different study, Jones found spikes in monthly account creations amongst users discussing Qatar on Twitter [43]. Jones then undertook the arduous task of manually reviewing a sample of almost ten thousand tweets along with their authors’ corresponding profile pictures and follower counts to find a worryingly large proportion of bots [43]. However, Jones did not explain whether multiple human coders were used during this analysis and how often they may have agreed or disagreed on their classifications of accounts as bots or humans. Furthermore, Jones did not examine the network structure of the data or perform a statistical comparison of the proportion of bots created during peak or non-peak times. To improve upon Jones’s manual analysis of a relatively small sample of tweets, we carried out an automated quantitative analysis of the phenomenon of account creation bursts at a large scale and further conducted statistical validation of the results. We also examined daily (rather than monthly) bursts in account creations, since at least one influence campaign analysis has reported the use of accounts created over a few days [40].

### Platform policies

In response to the threat posed by online influence operations, social media platforms have instituted algorithmic policies of not recommending potentially malicious content. Facebook states that it reduces distribution of content that its fact checking has determined to be false, in some cases going as far as to apply this reduction to all content from a user who has repeatedly shared false content [29]. Twitter has a similar stated policy of deranking undesirable content in its recommendation algorithms [30]. According to the company, it considers user behavior in making such decisions, including tracking whether “the same person signs up for multiple accounts simultaneously” [30]. Though the policy statement does not specify how Twitter determines if the same person is responsible for creating multiple accounts, it nevertheless makes clear that the platform aims to reduce the distribution of content posted by certain groups accounts that are created together. As our results show, however, detecting creation bursts can still identify suspicious accounts without considering user behavior or location.

## Methods

### Data collection

To investigate nefarious online activity during the 2020 U.S. elections, we used the Twitter streaming API to collect tweets about the elections from 1 December 2019 through 17 February 2021. This stream began by following hashtags and users involved in the Democratic presidential primaries and was modified over time to cover topics and users associated with post-primary campaigning and with contested Senate races. Finally, the stream was changed to focus on Biden’s inauguration. The details of these changes are specified in the Additional file 2: Methods. Including referenced tweets (such as retweeted and quoted tweets), we collected a total of 541,413,669 tweets authored by 21,114,981 users.

### Burst detection

At least one past analysis of an information operation has made reference to accounts being created over the span of a few days [40]. We therefore extracted all users’ creation times from our data and created a histogram of the number of users created each day. Because popular social media platforms naturally experience network effects in account creations during their early years of existence, we limited our analysis to accounts that were created at most ten years before the last day of data collection.

To detect bursts in account creations, we regarded the daily histogram of account creations as a one-dimensional signal and applied the cell-averaging constant false alarm rate (CA CFAR) algorithm [44], which uses a sensitivity parameter known as the probability of false alarm (PFA) to compute a daily threshold that, when exceeded by the signal, indicates a detection. In calculating this threshold for a given day, the algorithm averages the number of accounts created during a training window centered on the specified day, with the exception of the day itself and a certain number of days immediately surrounding it (known as guard cells). A constant factor involving the PFA is then applied to this average value to attain the final threshold for that day.

For our work, we used a training window of 30 days before and 30 days after a given day but removed the week centered on that day as guard cells. In other words, the three days surrounding a given day were excluded as guard cells, and the values of the remaining 54 days (27 days on either side of the given day) were averaged for calculating the threshold. Though this method uses information from after a given day’s occurrence, our work provides a proof of concept for the future study of other burst detection methods. Using our parameters and following the standard formulation of CA CFAR [44] yields Eq. 1 for computing the daily threshold, where $\varvec{h}$ is the vector of histogram values, *d* is the day for which the threshold is being calculated, $g=3$ is the number of guard cells on each side, and $t=27$ is the number of training cells on each side.

1 $$\begin{aligned} t_d = \sum _{i=g+1}^{g+t} \left( \varvec{h}_{d-i} + \varvec{h}_{d+i}\right) \left( {p_{fa}}^{\left( -\frac{1}{2t}\right) } - 1\right) \end{aligned}$$

Three PFA values—0.25, 0.30, and 0.35—were tested to highlight the parameter’s effect on our results. These values were chosen manually to showcase three different “kinds” of spikes in account creations: incredibly tall ones, shorter ones that to a human obviously still qualify as spikes, and even shorter ones that a human might think could be noise. Thus, though the least sensitive PFA setting of 0.25 likely captured bursts influenced by world events, the medium sensitivity setting yielded useful detections. It should also be noted that the bursts detected at higher sensitivities are supersets that include those detected at lower sensitivities. In other words, the bursts detected with a PFA of 0.30 include those detected with a PFA of 0.25, and the bursts detected with a PFA of 0.35 include those detected with a PFA of 0.30 or 0.25.

### Regression analysis

To test whether bursts of account creations were tied to phenomena that could indicate coordinated activity, we fitted separate regression models to various dependent variables reflecting these phenomena and examined the coefficients associated with the dummy (i.e., binary) independent variable indicating whether a data point came from a burst day or a non-burst day. Since our dependent variables were formulated as ranging from zero to one, our analysis made use of one-part fractional regression models [45], each implemented as a generalized linear model with quasibinomial family and logit link function [46]. This regression analysis was implemented in R version 4.0.5 using the “glm”, “coeftest”, and “margins” packages.

Since we only had the creation times for users involved in discussions of the 2020 elections, the sample of users was biased toward newer accounts and people who have used Twitter for a long time. This made it necessary to control for age when finding the relationship between account creation bursts and the dependent variables being modelled. The age of a data point was therefore added to the binary burst variable in each regression equation.

The level of analysis in each model was a day. In other words, each dependent variable had one value per day in the 10-year time period considered, with that value being based on the accounts created on that day. Since the dependent variables were calculated using information on all accounts created during the same day, modelling the data at the level of one day was necessary to maintain statistical independence between the data points.

For each coefficient in our fitted models, we performed *z*-tests with robust standard errors [47]. To quantify the magnitude of the relationship between account creation bursts and our various dependent variables, we calculated the average marginal effect [48] of each model’s binary burst variable by finding the mean difference in model predictions between the case in which all data points were assigned as bursts and the case in which all data points were assigned as non-bursts. In keeping with the coefficient analysis, we performed *z*-tests on the average marginal effects (AMEs) with robust standard errors estimated using the delta method [48].

Though all dependent variables could theoretically range from zero to one, some variables (such as network densities) had a natural tendency to have very small values. We therefore divided each AME by the standard deviation of the dependent variable to interpret the AME relative to the typical variability of that dependent variable. Since our analysis involved fitting one regression model per combination of dependent variable and PFA, we controlled for a false discovery rate of five percent by applying the Benjamini-Hochberg procedure [49] to the *z*-test *p*-values for the coefficients and AMEs of all independent and control variables.

### Network extraction

Several dependent variables developed for our regression analysis made use of the communication network between users along with their hashtag usage patterns. To derive the directed communication network between users, we weighted each network link with the number of tweets authored by one user that targeted another user through retweets, quotes, mentions, or replies to that other user. Each user’s hashtag choices were quantified by counting the number of tweets a user authored that contained a given hashtag. For both processes, we adopted the policy of attributing the contents of a retweet to both the retweeting and retweeted users. However, for tweets quoting other tweets, we attributed only the additional content to the quoting user and credited the quoted content to the quoted user. This accounted for the common practice of quoting a tweet even when expressing disagreement with that tweet.

### Dependent variables

#### Similarity of connections

To quantify the similarity users’ communication partners, we used the communication network to calculate the average cosine similarity of the neighborhoods of accounts created on the same day. We also computed the average local clustering coefficient [50] of users created on the same day. These mean neighborhood cosine similarities ($n =$ 3654, $\mu = 0.265$, $\sigma = 0.036$) and mean clustering coefficients ($n =$ 3,654, $\mu = 0.390$, $\sigma = 0.015$) were used as dependent variables in several regression models.

#### Density of contemporaries

To investigate whether users created together communicated with each other more often if they were created during a burst, we calculated the density of connections between accounts created on the same day. Using the communication network, we counted how many connections existed between the users created on a given day and divided by the total number of directed connections that could have existed (i.e., $n(n-1)$ for a set of *n* users). These density values ($n =$ 3,654, $\mu = 1\textrm{e}{-06}$, $\sigma = 2\textrm{e}{-06}$) were used as the dependent variable for a set of regression models.

#### Similarity of hashtag choices

We also examined whether accounts created during bursts tended to discuss more similar topics. After compiling the number of tweets each account authored containing a given hashtag, we calculated the average cosine similarity of these hashtag choices for accounts created on the same day. These daily hashtag similarities ($n =$ 3,654, $\mu = 0.007$, $\sigma = 0.005$) were then used as a dependent variable in our regression analysis.

#### User stance

To determine each user’s stance on political issues such as mail-in voting and mask wearing, we used the stance detector [51] built into version 3.0.9.126 of the ORA software [52]. This detector starts with a set of hashtags labeled as being in favor of or against a given issue and uses those hashtags to label the stances of the accounts that used them in their tweets. Influence propagation is then used to determine other users’ stances by repeating two steps. First, stance is spread from labeled users to connected (but unlabeled) users as well as to unlabeled hashtags used primarily by labeled accounts. Second, stance is spread from labeled hashtags and users to unlabeled users.

For our first run of this stance detection algorithm, we initialized the process with 30 hashtags in favor of and 30 hashtags against mail-in voting. The second run used 15 hashtags in favor of mask wearing and 15 hashtags against it. These hashtags are listed in Additional file 2: Methods. The stance confidence values generated by the stance detector were linearly remapped to range from zero (against) to one (in favor), with 0.5 representing a neutral stance. For each day in the 10-year time period, we calculated the standard deviation of the stance scores for accounts created on that day. The daily standard deviations of mail-in voting stances ($n =$ 3654, $\mu = 0.283$, $\sigma = 0.006$) and standard deviations of mask wearing stances ($n =$ 3654, $\mu = 0.263$, $\sigma = 0.006$) thereby quantified the level of disagreement on these issues amongst accounts created on the same day and were then used as dependent variables in separate sets of regression models.

#### Bot activity

Though many automated social media accounts are innocuous or even beneficial, social bots have also seen extensive use in online influence operations [10, 53]. For this reason, we sought to evaluate whether account creation bursts contained disproportionately more bots. To estimate the probability that an account is a bot, we used the Tier-1 BotHunter model, a random forest regressor that considers several features, including numbers of friends and followers, tweet content, and the timing of tweets [15]. The model showed an AUC of 0.994 on data about a 2017 attack against the Atlantic Council’s Digital Forensic Lab [15]. Using BotHunter, we computed the daily proportion of created users whose bot probabilities met or exceeded a threshold of 70%, which prior work has shown to provide more stable bot classifications when using a limited number of tweets per account [54]. These daily proportions ($n =$ 3654, $\mu = 0.152$, $\sigma = 0.052$) were then used as the dependent variable in a set of regression models.

#### Sharing of low-credibility sources

Given that not all online coordinated activity is malicious, we also studied users’ likelihood of sharing links to sites with low credibility. We extracted all URLs in our collected tweets, expanded them [55], and identified their top-level domains. We also developed an inexhaustive list of 954 Web domains belonging to sites that either (a) were listed as fake news by PolitiFact [56], (b) were included as black sites in the misinformation study by Grinberg *et al.* [57], or (c) were rated by Media Bias/Fact Check as being questionable or conspiracy sites with low or very low factual reporting [58, 59]. To this list, we added Breitbart and the *Daily Mail*, which are known purveyors of questionable stories [60]. The complete list can be found in Additional methods.

Using the expanded URLs’ domains and the list of questionable sites, we identified which users tweeted or retweeted at least one link to a low-credibility source. We then computed the proportion of accounts created on each day that eventually shared links to such sites. These proportions ($n =$ 3654, $\mu = 0.024$, $\sigma = 0.006$) were used as the dependent variable in a final set of regression models.

## Results

### Bursts of account creations

**Fig. 2:**
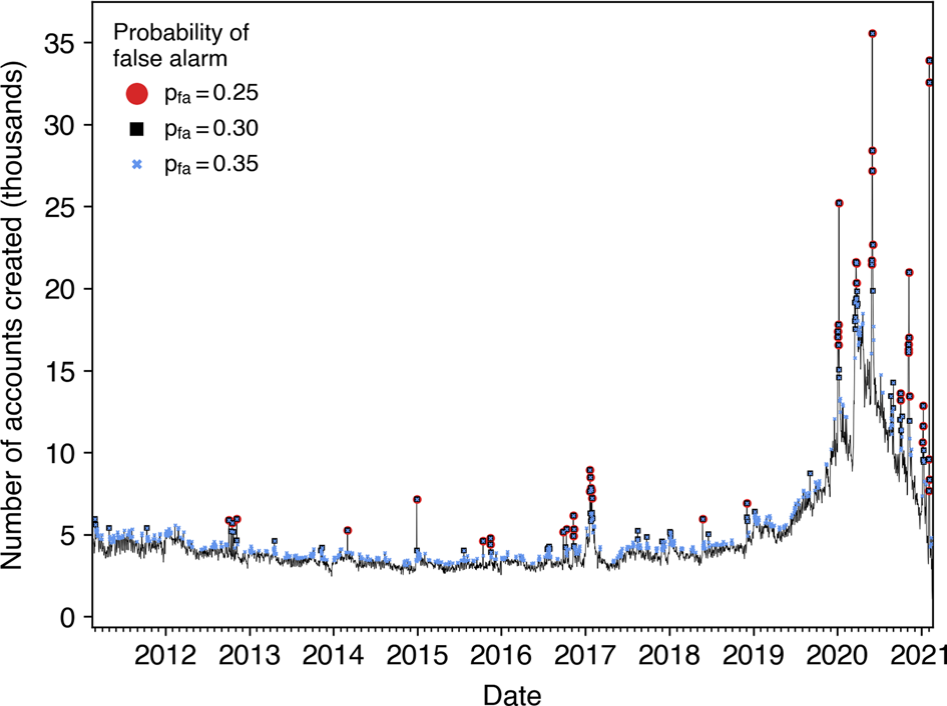
The number of Twitter users created each day that appeared in our collection of election tweets. Bursts detected using different CFAR sensitivities are also indicated using different symbols

When using the most sensitive PFA of 0.35, we detected 669 account creation bursts, whereas the CFAR algorithm detected 112 bursts with a PFA of 0.30 and 51 bursts with a PFA of 0.25. Figure 2 shows that the more restrictive PFA settings of 0.25 and 0.30 successfully isolated the most obvious bursts in account creations. The highest PFA additionally identified shorter spikes that would not be as conspicuous to a human. We can furthermore see a large increase in account creations during 2020, which is a necessary effect of having collected data primarily during 2020 on users discussing the U.S. elections.

### Communication with others

After isolating these bursts of account creations, we found that users’ communication with others, including their retweets, mentions, and replies, was more similar between accounts created on the same burst day than for accounts created on non-burst days. Even when controlling for the age of an account, fractional regression models [45, 46] fitted for predicting a given day’s average neighborhood similarity yielded significant positive coefficients for the variable indicating whether that day saw a burst in account creations.

Table 1 shows the AMEs calculated for the burst indicator variable in each of this study’s regression models. The complete model specifications are available in Additional file 1: Tables S1 to S24 results, and the AMEs for all variables are shown in Additional file 1: Tables S25 to S48. When detecting bursts with medium sensitivity, the neighborhood similarities of accounts created on burst days increased by an additional 1.11 times the overall standard deviation of neighborhood similarities, indicating a large relative increase in the homogeneity of these users’ communication patterns. For the tallest spikes detected with low sensitivity, this homogeneity was even greater, with an average increase of 1.38 standard deviations. Controlling for age showed that these differences were not solely an effect of whether the accounts had been created during our data collection period or had remained active for a long time.

**Table 1:** The average marginal effects (AME) of the binary burst variable on each indicator of coordination

PFA	0.25		0.30		0.35	
Dependent Var.	AME	$\frac{\text {AME}}{\sigma }$	AME	$\frac{\text {AME}}{\sigma }$	AME	$\frac{\text {AME}}{\sigma }$
Mean neighbor- hood similarity	0.050$$^{***}$$	1.38	0.040$$^{***}$$	1.11	0.017$$^{***}$$	0.46
Mean clustering coefficient	0.011$$^{*}$$	0.76	0.007$$^{**}$$	0.51	$$9\textrm{e}{-04}$$	0.06
Density amongst contemporaries	$$1\textrm{e}{-06}$$$$^{**}$$	0.62	$$9\textrm{e}{-07}$$$$^{***}$$	0.42	$$3\textrm{e}{-07}$$$$^{***}$$	0.15
Mean hashtag similarity	0.010	2.11	0.005$$^{*}$$	0.97	$$9\textrm{e}{-04}$$$$^{*}$$	0.19
Mail-in voting stance std. dev.	-0.016$$^{***}$$	-2.55	-0.009$$^{***}$$	-1.42	-0.002$$^{***}$$	-0.34
Mask wearing stance std. dev.	-0.011$$^{**}$$	-1.84	-0.005$$^{*}$$	-0.83	-0.001$$^{**}$$	-0.18
Prop. bots $$\ge $$ 70%	0.022$$^{***}$$	0.41	0.016$$^{***}$$	0.31	$$3\textrm{e}{-04}$$	$$6\textrm{e}{-03}$$
Prop. sharing low- credibility sites	0.003	0.53	0.004$$^{**}$$	0.73	0.002$$^{***}$$	0.41

Each AME was calculated as the average difference in model predictions between all data points being assigned as bursts and all data points being assigned as non-bursts. They were then standardized by dividing by the standard deviation $(\upsigma )$ of the dependent variable. Three asterisks indicate *p* < 0.001, two indicate *p* < 0.01, and one indicates *p* < 0.05. All *p*-values were corrected to control for a false discovery rate of 5%. Complete model specifications and AMEs for all variables are available in Additional file 1: Results

In addition, separate fractional regression models similarly indicated that accounts created during bursts tended to have more tightly knit communities surrounding them in the communication network. This average increase in mean clustering coefficient was also notable, at 0.51 standard deviations when using medium-sensitivity burst detection and 0.76 standard deviations when using the strictest detection. Greater clustering coefficients for burst accounts reveal that they tended to be more tightly embedded in their communities, some of which may have been echo chambers or bot nets.

### Talk amongst contemporaries

**Fig. 3:**
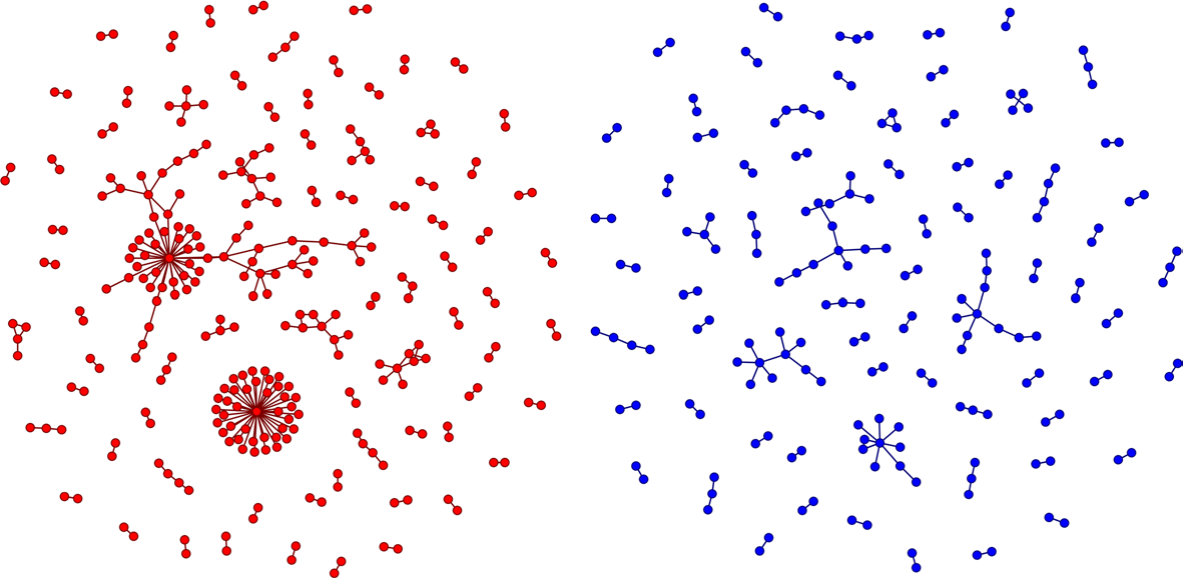
The communication networks for two days with similar numbers of created users: 6 January 2020 (left), a burst day, and 24 April 2020 (right), a non-burst day. Not shown are 16,261 users from 6 January and 16,089 users from 24 April who did not communicate with any other users created on their respective days

Additional regression models examining the density of connections between users created on the same day found that accounts created on burst days were also more likely to communicate amongst themselves. Figure 3 demonstrates the greater density of communication that these models show was tied to bursts of account creations. Though the average difference in raw density values was small, communication densities themselves tend to be extremely small. When compared to the standard deviation of these densities, accounts created on burst days exhibited an average increase of an additional 0.42 standard deviations when using medium-sensitivity burst detection and 0.62 standard deviations when using the least sensitive burst detection. This increased communication density makes it more likely that accounts created during bursts could have been coordinating their activity to, for example, construct echo chambers or bot nets.

### Hashtag usage patterns

We also observed that accounts created during medium or tall spikes tended to choose more similar sets of hashtags amongst themselves. Regression models showed that the average cosine similarity of hashtag usage by accounts created during the same burst day tended to be greater than for accounts created on non-burst days by almost an entire standard deviation when using medium-sensitivity burst detection. This effect was more than twice as large for bursts detected using the lowest sensitivity, but the *p*-value was not significant after false discovery rate correction.

The fact that the increase in hashtag similarity became not significant for the tallest spikes in account creations makes sense when considering the kind of activity that could produce such bursts. Several reports on influence campaigns [39–41] have revealed operations involving coordinated networks numbering in the hundreds of accounts, but the bursts detected using the most conservative PFA setting often involved increases of several thousands of accounts. This means that the largest bursts observed in Figure 2 likely involved significant “natural” activity inspired by world events. In fact, ten of the 51 bursts detected with a PFA of 0.25 occurred on the days following the U.S. elections in 2012, 2016, and 2020.

Account creation bursts were therefore tied to more similar hashtag use when discussing the 2020 elections, but only for bursts that were not so large that they could only have been produced with significant contribution from authentic activity. This makes it more likely that some such accounts could have been coordinating their messages to target the same topics.

### Agreement on political issues

Next, we examined whether accounts created during bursts tended to have more similar stances on the normally controversial issues of mail-in voting and mask wearing. For both mail-in voting and mask wearing, regression models fitted to our disagreement measurements yielded significant negative coefficients for the variable indicating whether a given day saw a burst of account creations.

On average, disagreement on mail-in voting decreased by 1.41 standard deviations amongst accounts created during bursts detected with medium sensitivity and by 2.55 standard deviations amongst accounts created during the tallest spikes. Similarly, disagreement on mask wearing fell by 0.83 standard deviations amongst accounts created during medium-sensitivity bursts and by 1.84 standard deviations for those created during low-sensitivity bursts. These account creation bursts were therefore tied to significantly smaller standard deviations in user stance, indicating that there was disproportionately more agreement between these users on these controversial issues. This result adds to the suspicion that some of the accounts created during bursts may have been set up to support certain causes in a coordinated manner.

### Bot activity

Notably, we found that burst days tended to see comparatively more bots being created. On average, 1.6% more bots were created during bursts detected with medium sensitivity, which represented an increase of 0.31 standard deviations in the proportion of bots. The largest bursts saw 2.2% more bots created, an increase of 0.41 standard deviations. Though these effect sizes are smaller than for the other results, one should recall that the analysis used a conservatively high threshold for detecting bots. Though past work [54] has shown this threshold to perform best for identifying bots using small numbers of tweets, it is likely that using a high threshold missed some bots.

### Sharing of low-credibility sources

Though increased similarity in user behavior suggests a greater likelihood of coordination in general, we further found that these increased indicators of coordination were tied to more sharing of misinformation, suggesting that this coordination may have been malicious. Corresponding regression models showed that an account created during a burst detected with medium or high sensitivity was significantly more likely to eventually share a link to a low-credibility source. For medium-sensitivity bursts, this likelihood increased by 0.73 standard deviations.

However, increased sharing to low-credibility sources was not significant for the tallest spikes in account creations detected using the lowest sensitivity. Like with the results on hashtag similarity, this can be explained by the significant amount of authentic account creations required to produce bursts numbering in the several thousands of accounts rather than the hundreds reportedly created together for information operations [39–41]. Nevertheless, account creation bursts of a size that could have been caused by inauthentic activity were tied to greater sharing of sites with questionable credibility, increasing the chance that such accounts were malicious.

## Discussion

Ideally, social media companies would identify and thwart malicious actors as soon as possible. However, such rapid decision-making naturally comes at the expense of having less information available when deciding whether to intervene. This is of particularly serious concern given that social media interventions can be used to disrupt legitimate forms of coordination like protests [61]. When available information is insufficient to confidently decide to suspend an account but the actor still appears to be suspicious, “softer” measures for countering possible influence campaigns should be preferred.

Our work has shown how the very act of online account creation can draw immediate suspicion upon sets of possibly malicious actors. Accounts created in unnatural bursts were tied to increased similarity in behavior and political stance as well as greater likelihoods of being automated and sharing links to low-credibility sites. Detecting these bursts to swiftly identify suspicious accounts could therefore assist social media platforms in pre-emptively hindering the dissemination of misinformation, which can be extremely valuable considering the rapidity with which it spreads [62]. However, like with previous work examining suspicious account creations [38], the burst detection presented by this study cannot be used in isolation because of the legitimate accounts that would be caught in the crossfire. Rather, we advocate for a shift in the thinking currently associated with research on misinformation and social media interventions.

To keep pace with the constant changes in malicious actors’ online strategies, we join others in believing that scholars and professionals would be best served by developing interoperable tools and methodologies [28] that can be used by social media companies to gradually build evidence of harmfulness against actors and incrementally hamper their efforts as the suspicions against them are substantiated. Just as countries can sanction foreign entities to varying levels of severity, so too can social media platforms incrementally sanction bad actors in ways other than outright suspension. Since the companies themselves control the algorithms used to recommend content to their platforms’ users, they are free to penalize certain content in these recommendation systems. Social media companies are obviously aware of this, already implementing policies to derank messages that have been deemed false as well as content from users exhibiting suspicious behavior [29, 30]. However, published research in the area of social cybersecurity has so far focused on individual detection systems with little effort to integrate them [28].

By detecting sudden bursts of account creations, social media companies would have the opportunity to immediately penalize and investigate questionable accounts until a final determination can be made to suspend them or clear them of suspicion. These companies could further improve this strategy by implementing and investigating more advanced burst detection strategies. In concert with evidence produced by other tools and methods for detecting misinformation campaigns, the identification of account creation bursts could thereby help mitigate the effects of future attacks on democratic societies and ensure greater social cybersecurity. Given the promising potential of integrating multiple anti-misinformation methods for countering malicious actors’ constantly changing strategies, we believe researchers should strive to develop more integrable methods for identifying bad actors and harmful content on social media.

## Conclusions

Using the largest fully analyzed Twitter data set on the 2020 U.S. elections to date, we discovered abnormal spikes in the daily numbers of account creations and found these bursts to be associated with heightened indicators of possibly malicious coordination. Compared to other accounts, those created during these bursts exhibited more similar behavior, agreed more on normally controversial issues, were more likely to be bots, and were more likely to share links to low-credibility sites. Though classifying accounts based solely on their creation date is not a viable way of conclusively determining whether to suspend them, social media companies could pre-emptively limit their influence by incorporating this strategy into existing platform policies for deranking content [29, 30].

If combined with other methods for detecting coordinated malicious activity, tracking account creation bursts would help platforms hinder coordinated influence campaigns from their very beginning, even before inauthentic accounts display undesirable behavior. Given the importance of stopping malicious content as early as possible, this would contribute to promoting social cybersecurity in democracies threatened by information operations. Given the advantages associated with combining multiple approaches for countering social media manipulation, we join others [28] in calling upon the research community to develop more interoperable methods for combatting online influence campaigns.

## Supplementary Information

**Additional file 1.** Additional results.

**Additional file 2.** Additional methods.

## Data Availability

Because of issues relating to user privacy, Twitter’s policies, and data size, only the derived variables used in the regression analysis are available from the authors upon reasonable request and with approval from the research sponsors.

## Abbreviations

PFA: Probability of False Alarm
AME: Average Marginal Effect
CA CFAR: Cell-Averaging Constant False Alarm Rate
API: Application Programming Interface
U.S.: United States
