# Supplementary material for: Investigating coordinated account creation using burst detection and network analysis
Source: J Big Data. 2023 Feb 10;10(1):20. doi: 10.1186/s40537-023-00695-7 (PMC9913025; doi:10.1186/s40537-023-00695-7)
Supplement: Supplementary file 1 — Additional file 1. Additional results. [file 40537_2023_695_MOESM1_ESM.pdf]

# Supplementary results for “Investigating coordinated account creation using burst detection and network analysis”

Daniele Bellutta & Kathleen M. Carley

## Similarity of behavior

### Communication with others

Tables 1 through 3 contain the complete specifications for the regression models fit to predict daily average neighborhood similarities, whereas Tables 25 through 27 contain information on the corresponding average marginal effects. Tables 4 through 6 contain the complete specifications for the regression models fit to predict daily average clustering coefficients, whereas Tables 28 through 30 contain information on the corresponding average marginal effects.

### Talk amongst contemporaries

Tables 7 through 9 contain the complete specifications for the regression models fit to predict the density of communication between users created on each day, whereas Tables 31 through 33 contain information on the corresponding average marginal effects.

### Hashtag use

Tables 10 through 12 contain the complete specifications for the regression models fit to predict the average hashtag similarities between users created on each day, whereas Tables 34 through 36 contain information on the corresponding average marginal effects.

## Agreement on political issues

Tables 13 through 15 contain the complete specifications for the regression models fit to predict agreement on mail-in voting amongst users created on each day, whereas Tables 37 through 39 contain information on the corresponding average marginal effects. Tables 16 through 18 contain the complete specifications for the regression models fit to predict agreement on mask wearing amongst users created on each day, whereas Tables 40 through 42 contain information on the corresponding average marginal effects.

## Bot activity

Tables 19 through 21 contain the complete specifications for the regression models fit to predict the daily proportions of created users with bot scores greater than or equal to 70%, whereas Tables 43 through 45 contain information on the corresponding average marginal effects.

## Sharing of low-credibility sources

Tables 22 through 24 contain the complete specifications for the regression models fit to predict the daily proportions of created users who shared at least one link to a low-credibility site, whereas Tables 46 through 48 contain information on the corresponding average marginal effects.

Table 1: Regression coefficients when modelling daily mean of created users' neighborhood cosine similarities, as computed with low-sensitivity bursts.

| Variable | Coefficient | SE | $z$ | $p$ | Corrected $p$ |
|---|---|---|---|---|---|
| Intercept | −0.939623 | 0.009106 | −103.191966 | 0e+00 | |
| Burst | 0.241688 | 0.052452 | 4.607789 | 0.000004 | 0.000009 |
| Age | −0.017155 | 0.001341 | −12.791957 | 2e−37 | 1e−36 |
| $N$ | 3654 | | | | |
| Fit RMSD | 0.033950 | | | | |
| $\frac{\text{RMSD}}{\sigma}$ | 0.944295 | | | | |

The complete specification for a one-part fractional regression model fit to predict each day's mean neighborhood cosine similarity amongst accounts created on that day. Account creation bursts were detected with a probability of false alarm (PFA) of 0.25. Each variable coefficient is provided, along with its robust standard error (SE), $z$-score, raw $p$-value, and corrected $p$-value. The number of data points ($N$), the root-mean-square deviation (RMSD) achieved on the fitted data, and the RMSD divided by the standard deviation of the dependent variable $\left(\frac{\text{RMSD}}{\sigma}\right)$ have also been provided.

Table 2: Regression coefficients when modelling daily mean of created users' neighborhood cosine similarities, as computed with medium-sensitivity bursts.

| Variable | Coefficient | SE | $z$ | $p$ | Corrected $p$ |
|---|---|---|---|---|---|
| Intercept | −0.944542 | 0.008949 | −105.543472 | 0e+00 | |
| Burst | 0.197605 | 0.033653 | 5.871783 | 4e−09 | 1e−08 |
| Age | −0.016725 | 0.001322 | −12.653784 | 1e−36 | 6e−36 |
| $N$ | 3654 | | | | |
| Fit RMSD | 0.033749 | | | | |
| $\frac{\text{RMSD}}{\sigma}$ | 0.938708 | | | | |

The complete specification for a one-part fractional regression model fit to predict each day's mean neighborhood cosine similarity amongst accounts created on that day. Account creation bursts were detected with a probability of false alarm (PFA) of 0.30. Each variable coefficient is provided, along with its robust standard error (SE), $z$-score, raw $p$-value, and corrected $p$-value. The number of data points ($N$), the root-mean-square deviation (RMSD) achieved on the fitted data, and the RMSD divided by the standard deviation of the dependent variable $\left(\frac{\text{RMSD}}{\sigma}\right)$ have also been provided.

Table 3: Regression coefficients when modelling daily mean of created users' neighborhood cosine similarities, as computed with high-sensitivity bursts.

| Variable | Coefficient | SE | $z$ | $p$ | Corrected $p$ |
|---|---|---|---|---|---|
| Intercept | −0.947639 | 0.009050 | −104.708522 | 0e+00 | |
| Burst | 0.084748 | 0.009007 | 9.409533 | 5e−21 | 3e−20 |
| Age | −0.017996 | 0.001345 | −13.381445 | 8e−41 | 6e−40 |
| $N$ | 3654 | | | | |
| Fit RMSD | 0.033829 | | | | |
| $\frac{\text{RMSD}}{\sigma}$ | 0.940918 | | | | |

The complete specification for a one-part fractional regression model fit to predict each day's mean neighborhood cosine similarity amongst accounts created on that day. Account creation bursts were detected with a probability of false alarm (PFA) of 0.35. Each variable coefficient is provided, along with its robust standard error (SE), $z$-score, raw $p$-value, and corrected $p$-value. The number of data points ($N$), the root-mean-square deviation (RMSD) achieved on the fitted data, and the RMSD divided by the standard deviation of the dependent variable $\left(\frac{\text{RMSD}}{\sigma}\right)$ have also been provided.

Table 4: Regression coefficients when modelling daily mean of created users' clustering coefficients, as computed with low-sensitivity bursts.

| Variable | Coefficient | SE | $z$ | $p$ | Corrected $p$ |
|---|---|---|---|---|---|
| Intercept | −0.380297 | 0.002135 | −178.127880 | 0e+00 | |
| Burst | 0.046281 | 0.017987 | 2.573000 | 0.010082 | 0.013259 |
| Age | −0.013557 | 0.000316 | −42.898436 | 0e+00 | 0e+00 |
| $N$ | 3654 | | | | |
| Fit RMSD | 0.010998 | | | | |
| $\frac{\text{RMSD}}{\sigma}$ | 0.754211 | | | | |

The complete specification for a one-part fractional regression model fit to predict each day's mean clustering coefficient for accounts created on that day. Account creation bursts were detected with a probability of false alarm (PFA) of 0.25. Each variable coefficient is provided, along with its robust standard error (SE), $z$-score, raw $p$-value, and corrected $p$-value. The number of data points ($N$), the root-mean-square deviation (RMSD) achieved on the fitted data, and the RMSD divided by the standard deviation of the dependent variable $\left(\frac{\text{RMSD}}{\sigma}\right)$ have also been provided.

Table 5: Regression coefficients when modelling daily mean of created users' clustering coefficients, as computed with medium-sensitivity bursts.

| Variable | Coefficient | SE | $z$ | $p$ | Corrected $p$ |
|---|---|---|---|---|---|
| Intercept | −0.380751 | 0.002123 | −179.312128 | 0e+00 | |
| Burst | 0.031065 | 0.009776 | 3.177553 | 0.001485 | 0.002300 |
| Age | −0.013527 | 0.000315 | −42.893349 | 0e+00 | 0e+00 |
| $N$ | 3654 | | | | |
| Fit RMSD | 0.011001 | | | | |
| $\frac{\text{RMSD}}{\sigma}$ | 0.754395 | | | | |

The complete specification for a one-part fractional regression model fit to predict each day's mean clustering coefficient for accounts created on that day. Account creation bursts were detected with a probability of false alarm (PFA) of 0.30. Each variable coefficient is provided, along with its robust standard error (SE), $z$-score, raw $p$-value, and corrected $p$-value. The number of data points ($N$), the root-mean-square deviation (RMSD) achieved on the fitted data, and the RMSD divided by the standard deviation of the dependent variable $\left(\frac{\text{RMSD}}{\sigma}\right)$ have also been provided.

Table 6: Regression coefficients when modelling daily mean of created users' clustering coefficients, as computed with high-sensitivity bursts.

| Variable | Coefficient | SE | $z$ | $p$ | Corrected $p$ |
|---|---|---|---|---|---|
| Intercept | −0.379359 | 0.002173 | −174.577943 | 0e+00 | |
| Burst | 0.003714 | 0.002466 | 1.506123 | 0.132036 | 0.158589 |
| Age | −0.013750 | 0.000331 | −41.581123 | 0e+00 | 0e+00 |
| $N$ | 3654 | | | | |
| Fit RMSD | 0.011069 | | | | |
| $\frac{\text{RMSD}}{\sigma}$ | 0.759110 | | | | |

The complete specification for a one-part fractional regression model fit to predict each day's mean clustering coefficient for accounts created on that day. Account creation bursts were detected with a probability of false alarm (PFA) of 0.35. Each variable coefficient is provided, along with its robust standard error (SE), $z$-score, raw $p$-value, and corrected $p$-value. The number of data points ($N$), the root-mean-square deviation (RMSD) achieved on the fitted data, and the RMSD divided by the standard deviation of the dependent variable $\left(\frac{\text{RMSD}}{\sigma}\right)$ have also been provided.

Table 7: Regression coefficients when modelling density of communication between users created on each day, as computed with low-sensitivity bursts.

| Variable | Coefficient | SE | $z$ | $p$ | Corrected $p$ |
|---|---|---|---|---|---|
| Intercept | −13.467652 | 0.055090 | −244.467578 | 0e+00 | |
| Burst | 0.747510 | 0.181863 | 4.110282 | 0.000040 | 0.000070 |
| Age | −0.041269 | 0.008267 | −4.992077 | 6e−07 | 0.000002 |
| $N$ | 3654 | | | | |
| Fit RMSD | 0.000002 | | | | |
| $\frac{\text{RMSD}}{\sigma}$ | 0.993878 | | | | |

The complete specification for a one-part fractional regression model fit to predict the communication density between the users created on each day. Account creation bursts were detected with a probability of false alarm (PFA) of 0.25. Each variable coefficient is provided, along with its robust standard error (SE), $z$-score, raw $p$-value, and corrected $p$-value. The number of data points ($N$), the root-mean-square deviation (RMSD) achieved on the fitted data, and the RMSD divided by the standard deviation of the dependent variable $\left(\frac{\text{RMSD}}{\sigma}\right)$ have also been provided.

Table 8: Regression coefficients when modelling density of communication between users created on each day, as computed with medium-sensitivity bursts.

| Variable | Coefficient | SE | $z$ | $p$ | Corrected $p$ |
|---|---|---|---|---|---|
| Intercept | −13.479059 | 0.056545 | −238.375697 | 0e+00 | |
| Burst | 0.565231 | 0.120409 | 4.694262 | 0.000003 | 0.000006 |
| Age | −0.040507 | 0.008404 | −4.820098 | 0.000001 | 0.000004 |
| $N$ | 3654 | | | | |
| Fit RMSD | 0.000002 | | | | |
| $\frac{\text{RMSD}}{\sigma}$ | 0.994084 | | | | |

The complete specification for a one-part fractional regression model fit to predict the communication density between the users created on each day. Account creation bursts were detected with a probability of false alarm (PFA) of 0.30. Each variable coefficient is provided, along with its robust standard error (SE), $z$-score, raw $p$-value, and corrected $p$-value. The number of data points ($N$), the root-mean-square deviation (RMSD) achieved on the fitted data, and the RMSD divided by the standard deviation of the dependent variable $\left(\frac{\text{RMSD}}{\sigma}\right)$ have also been provided.

Table 9: Regression coefficients when modelling density of communication between users created on each day, as computed with high-sensitivity bursts.

| Variable | Coefficient | SE | $z$ | $p$ | Corrected $p$ |
|---|---|---|---|---|---|
| Intercept | −13.483481 | 0.059789 | −225.517495 | 0e+00 | |
| Burst | 0.253917 | 0.057777 | 4.394755 | 0.000011 | 0.000023 |
| Age | −0.045362 | 0.008421 | −5.386634 | 7e−08 | 2e−07 |
| $N$ | 3654 | | | | |
| Fit RMSD | 0.000002 | | | | |
| $\frac{\text{RMSD}}{\sigma}$ | 0.995120 | | | | |

The complete specification for a one-part fractional regression model fit to predict the communication density between the users created on each day. Account creation bursts were detected with a probability of false alarm (PFA) of 0.35. Each variable coefficient is provided, along with its robust standard error (SE), $z$-score, raw $p$-value, and corrected $p$-value. The number of data points ($N$), the root-mean-square deviation (RMSD) achieved on the fitted data, and the RMSD divided by the standard deviation of the dependent variable $\left(\frac{\text{RMSD}}{\sigma}\right)$ have also been provided.

Table 10: Regression coefficients when modelling daily mean of created users' hashtag cosine similarities, as computed with low-sensitivity bursts.

| Variable | Coefficient | SE | $z$ | $p$ | Corrected $p$ |
|---|---|---|---|---|---|
| Intercept | −5.044978 | 0.023656 | −213.261896 | 0e+00 | |
| Burst | 0.928195 | 0.299209 | 3.102161 | 0.001921 | 0.002882 |
| Age | 0.004245 | 0.003986 | 1.064983 | 0.286884 | 0.320242 |
| $N$ | 3654 | | | | |
| Fit RMSD | 0.004534 | | | | |
| $\frac{\text{RMSD}}{\sigma}$ | 0.970439 | | | | |

The complete specification for a one-part fractional regression model fit to predict each day's mean hashtag cosine similarity amongst accounts created on that day. Account creation bursts were detected with a probability of false alarm (PFA) of 0.25. Each variable coefficient is provided, along with its robust standard error (SE), $z$-score, raw $p$-value, and corrected $p$-value. The number of data points ($N$), the root-mean-square deviation (RMSD) achieved on the fitted data, and the RMSD divided by the standard deviation of the dependent variable $\left(\frac{\text{RMSD}}{\sigma}\right)$ have also been provided.

Table 11: Regression coefficients when modelling daily mean of created users' hashtag cosine similarities, as computed with medium-sensitivity bursts.

| Variable | Coefficient | SE | $z$ | $p$ | Corrected $p$ |
|---|---|---|---|---|---|
| Intercept | −5.038551 | 0.026311 | −191.496349 | 0e+00 | |
| Burst | 0.532626 | 0.201521 | 2.643030 | 0.008217 | 0.011091 |
| Age | 0.002886 | 0.004585 | 0.629455 | 0.529051 | 0.564321 |
| $N$ | 3654 | | | | |
| Fit RMSD | 0.004608 | | | | |
| $\frac{\text{RMSD}}{\sigma}$ | 0.986266 | | | | |

The complete specification for a one-part fractional regression model fit to predict each day's mean hashtag cosine similarity amongst accounts created on that day. Account creation bursts were detected with a probability of false alarm (PFA) of 0.30. Each variable coefficient is provided, along with its robust standard error (SE), $z$-score, raw $p$-value, and corrected $p$-value. The number of data points ($N$), the root-mean-square deviation (RMSD) achieved on the fitted data, and the RMSD divided by the standard deviation of the dependent variable $\left(\frac{\text{RMSD}}{\sigma}\right)$ have also been provided.

Table 12: Regression coefficients when modelling daily mean of created users' hashtag cosine similarities, as computed with high-sensitivity bursts.

| Variable | Coefficient | SE | $z$ | $p$ | Corrected $p$ |
|---|---|---|---|---|---|
| Intercept | −5.019228 | 0.034176 | −146.865852 | 0e+00 | |
| Burst | 0.130817 | 0.053937 | 2.425339 | 0.015294 | 0.019576 |
| Age | −0.001799 | 0.006578 | −0.273395 | 0.784550 | 0.801903 |
| $N$ | 3654 | | | | |
| Fit RMSD | 0.004658 | | | | |
| $\frac{\text{RMSD}}{\sigma}$ | 0.996975 | | | | |

The complete specification for a one-part fractional regression model fit to predict each day's mean hashtag cosine similarity amongst accounts created on that day. Account creation bursts were detected with a probability of false alarm (PFA) of 0.35. Each variable coefficient is provided, along with its robust standard error (SE), $z$-score, raw $p$-value, and corrected $p$-value. The number of data points ($N$), the root-mean-square deviation (RMSD) achieved on the fitted data, and the RMSD divided by the standard deviation of the dependent variable $\left(\frac{\text{RMSD}}{\sigma}\right)$ have also been provided.

Table 13: Regression coefficients when modelling standard deviation of mail-in voting stances of users created on each day, as computed with low-sensitivity bursts.

| Variable | Coefficient | SE | $z$ | $p$ | Corrected $p$ |
|---|---|---|---|---|---|
| Intercept | −0.933589 | 0.001417 | −658.732848 | 0e+00 | |
| Burst | −0.082691 | 0.019757 | −4.185366 | 0.000028 | 0.000052 |
| Age | 0.000819 | 0.000221 | 3.695522 | 0.000219 | 0.000376 |
| $N$ | 3654 | | | | |
| Fit RMSD | 0.006127 | | | | |
| $\frac{\text{RMSD}}{\sigma}$ | 0.948729 | | | | |

The complete specification for a one-part fractional regression model fit to predict each day's standard deviation in mail-in voting stances amongst accounts created on that day. Account creation bursts were detected with a probability of false alarm (PFA) of 0.25. Each variable coefficient is provided, along with its robust standard error (SE), $z$-score, raw $p$-value, and corrected $p$-value. The number of data points ($N$), the root-mean-square deviation (RMSD) achieved on the fitted data, and the RMSD divided by the standard deviation of the dependent variable $\left(\frac{\text{RMSD}}{\sigma}\right)$ have also been provided.

Table 14: Regression coefficients when modelling standard deviation of mail-in voting stances of users created on each day, as computed medium-sensitivity bursts.

| Variable | Coefficient | SE | $z$ | $p$ | Corrected $p$ |
|---|---|---|---|---|---|
| Intercept | −0.933435 | 0.001466 | −636.692188 | 0e+00 | |
| Burst | −0.045515 | 0.009444 | −4.819523 | 0.000001 | 0.000004 |
| Age | 0.000837 | 0.000231 | 3.626332 | 0.000287 | 0.000468 |
| $N$ | 3654 | | | | |
| Fit RMSD | 0.006227 | | | | |
| $\frac{\text{RMSD}}{\sigma}$ | 0.964288 | | | | |

The complete specification for a one-part fractional regression model fit to predict each day's standard deviation in mail-in voting stances amongst accounts created on that day. Account creation bursts were detected with a probability of false alarm (PFA) of 0.30. Each variable coefficient is provided, along with its robust standard error (SE), $z$-score, raw $p$-value, and corrected $p$-value. The number of data points ($N$), the root-mean-square deviation (RMSD) achieved on the fitted data, and the RMSD divided by the standard deviation of the dependent variable $\left(\frac{\text{RMSD}}{\sigma}\right)$ have also been provided.

Table 15: Regression coefficients when modelling standard deviation of mail-in voting stances of users created on each day, as computed high-sensitivity bursts.

| Variable | Coefficient | SE | $z$ | $p$ | Corrected $p$ |
|---|---|---|---|---|---|
| Intercept | −0.934328 | 0.001564 | −597.305554 | 0e+00 | |
| Burst | −0.010969 | 0.002050 | −5.350109 | 9e−08 | 3e−07 |
| Age | 0.001140 | 0.000264 | 4.315924 | 0.000016 | 0.000031 |
| $N$ | 3654 | | | | |
| Fit RMSD | 0.006363 | | | | |
| $\frac{\text{RMSD}}{\sigma}$ | 0.985239 | | | | |

The complete specification for a one-part fractional regression model fit to predict each day's standard deviation in mail-in voting stances amongst accounts created on that day. Account creation bursts were detected with a probability of false alarm (PFA) of 0.35. Each variable coefficient is provided, along with its robust standard error (SE), $z$-score, raw $p$-value, and corrected $p$-value. The number of data points ($N$), the root-mean-square deviation (RMSD) achieved on the fitted data, and the RMSD divided by the standard deviation of the dependent variable $\left(\frac{\text{RMSD}}{\sigma}\right)$ have also been provided.

Table 16: Regression coefficients when modelling standard deviation of mask wearing stances of users created on each day, as computed with low-sensitivity bursts.

| Variable | Coefficient | SE | $z$ | $p$ | Corrected $p$ |
|---|---|---|---|---|---|
| Intercept | −1.021737 | 0.001473 | −693.452742 | 0e+00 | |
| Burst | −0.056760 | 0.020746 | −2.735946 | 0.006220 | 0.008912 |
| Age | −0.002047 | 0.000228 | −8.967588 | 3e−19 | 1e−18 |
| $N$ | 3654 | | | | |
| Fit RMSD | 0.005662 | | | | |
| $\frac{\text{RMSD}}{\sigma}$ | 0.961260 | | | | |

The complete specification for a one-part fractional regression model fit to predict each day's standard deviation in mask wearing stances amongst accounts created on that day. Account creation bursts were detected with a probability of false alarm (PFA) of 0.25. Each variable coefficient is provided, along with its robust standard error (SE), $z$-score, raw $p$-value, and corrected $p$-value. The number of data points ($N$), the root-mean-square deviation (RMSD) achieved on the fitted data, and the RMSD divided by the standard deviation of the dependent variable $\left(\frac{\text{RMSD}}{\sigma}\right)$ have also been provided.

Table 17: Regression coefficients when modelling standard deviation of mask wearing stances of users created on each day, as computed with medium-sensitivity bursts.

| Variable | Coefficient | SE | $z$ | $p$ | Corrected $p$ |
|---|---|---|---|---|---|
| Intercept | −1.022026 | 0.001528 | −668.665448 | 0e+00 | |
| Burst | −0.025465 | 0.009650 | −2.638864 | 0.008318 | 0.011091 |
| Age | −0.001990 | 0.000239 | −8.335742 | 8e−17 | 3e−16 |
| $N$ | 3654 | | | | |
| Fit RMSD | 0.005741 | | | | |
| $\frac{\text{RMSD}}{\sigma}$ | 0.974743 | | | | |

The complete specification for a one-part fractional regression model fit to predict each day's standard deviation in mask wearing stances amongst accounts created on that day. Account creation bursts were detected with a probability of false alarm (PFA) of 0.30. Each variable coefficient is provided, along with its robust standard error (SE), $z$-score, raw $p$-value, and corrected $p$-value. The number of data points ($N$), the root-mean-square deviation (RMSD) achieved on the fitted data, and the RMSD divided by the standard deviation of the dependent variable $\left(\frac{\text{RMSD}}{\sigma}\right)$ have also been provided.

Table 18: Regression coefficients when modelling standard deviation of mask wearing stances of users created on each day, as computed with high-sensitivity bursts.

| Variable | Coefficient | SE | $z$ | $p$ | Corrected $p$ |
|---|---|---|---|---|---|
| Intercept | −1.022670 | 0.001612 | −634.460486 | 0e+00 | |
| Burst | −0.005450 | 0.002001 | −2.724299 | 0.006444 | 0.008965 |
| Age | −0.001818 | 0.000270 | −6.722029 | 2e−11 | 6e−11 |
| $N$ | 3654 | | | | |
| Fit RMSD | 0.005788 | | | | |
| $\frac{\text{RMSD}}{\sigma}$ | 0.982712 | | | | |

The complete specification for a one-part fractional regression model fit to predict each day's standard deviation in mask wearing stances amongst accounts created on that day. Account creation bursts were detected with a probability of false alarm (PFA) of 0.35. Each variable coefficient is provided, along with its robust standard error (SE), $z$-score, raw $p$-value, and corrected $p$-value. The number of data points ($N$), the root-mean-square deviation (RMSD) achieved on the fitted data, and the RMSD divided by the standard deviation of the dependent variable $\left(\frac{\text{RMSD}}{\sigma}\right)$ have also been provided.

Table 19: Regression coefficients when modelling daily proportion of users created with bot scores ≥ 70%, as computed with low-sensitivity bursts.

| Variable | Coefficient | SE | $z$ | $p$ | Corrected $p$ |
|---|---|---|---|---|---|
| Intercept | −1.198462 | 0.011793 | −101.623192 | 0e+00 | |
| Burst | 0.161144 | 0.032017 | 5.033061 | 5e−07 | 0.000001 |
| Age | −0.111096 | 0.001812 | −61.317141 | 0e+00 | 0e+00 |
| $N$ | 3654 | | | | |
| Fit RMSD | 0.031500 | | | | |
| $\frac{\text{RMSD}}{\sigma}$ | 0.601400 | | | | |

The complete specification for a one-part fractional regression model fit to predict each day's proportion of created users with bot scores of at least 70%. Account creation bursts were detected with a probability of false alarm (PFA) of 0.25. Each variable coefficient is provided, along with its robust standard error (SE), $z$-score, raw $p$-value, and corrected $p$-value. The number of data points ($N$), the root-mean-square deviation (RMSD) achieved on the fitted data, and the RMSD divided by the standard deviation of the dependent variable $\left(\frac{\text{RMSD}}{\sigma}\right)$ have also been provided.

Table 20: Regression coefficients when modelling daily proportion of users created with bot scores ≥ 70%, as computed with medium-sensitivity bursts.

| Variable | Coefficient | SE | $z$ | $p$ | Corrected $p$ |
|---|---|---|---|---|---|
| Intercept | −1.201126 | 0.011812 | −101.687455 | 0e+00 | |
| Burst | 0.122692 | 0.025741 | 4.766477 | 0.000002 | 0.000004 |
| Age | −0.110871 | 0.001808 | −61.317667 | 0e+00 | 0e+00 |
| $N$ | 3654 | | | | |
| Fit RMSD | 0.031452 | | | | |
| $\frac{\text{RMSD}}{\sigma}$ | 0.600500 | | | | |

The complete specification for a one-part fractional regression model fit to predict each day's proportion of created users with bot scores of at least 70%. Account creation bursts were detected with a probability of false alarm (PFA) of 0.30. Each variable coefficient is provided, along with its robust standard error (SE), $z$-score, raw $p$-value, and corrected $p$-value. The number of data points ($N$), the root-mean-square deviation (RMSD) achieved on the fitted data, and the RMSD divided by the standard deviation of the dependent variable $\left(\frac{\text{RMSD}}{\sigma}\right)$ have also been provided.

Table 21: Regression coefficients when modelling daily proportion of users created with bot scores ≥ 70%, as computed with high-sensitivity bursts.

| Variable | Coefficient | SE | $z$ | $p$ | Corrected $p$ |
|---|---|---|---|---|---|
| Intercept | −1.192450 | 0.011720 | −101.743518 | 0e+00 | |
| Burst | 0.002606 | 0.011212 | 0.232403 | 0.816225 | 0.816330 |
| Age | −0.111933 | 0.001785 | −62.710904 | 0e+00 | 0e+00 |
| $N$ | 3654 | | | | |
| Fit RMSD | 0.031669 | | | | |
| $\frac{\text{RMSD}}{\sigma}$ | 0.604641 | | | | |

The complete specification for a one-part fractional regression model fit to predict each day's proportion of created users with bot scores of at least 70%. Account creation bursts were detected with a probability of false alarm (PFA) of 0.35. Each variable coefficient is provided, along with its robust standard error (SE), $z$-score, raw $p$-value, and corrected $p$-value. The number of data points ($N$), the root-mean-square deviation (RMSD) achieved on the fitted data, and the RMSD divided by the standard deviation of the dependent variable $\left(\frac{\text{RMSD}}{\sigma}\right)$ have also been provided.

Table 22: Regression coefficients when modelling daily proportion of created users sharing low-credibility sites, as computed with low-sensitivity bursts.

| Variable | Coefficient | SE | $z$ | $p$ | Corrected $p$ |
|---|---|---|---|---|---|
| Intercept | −3.689571 | 0.008709 | −423.643867 | 0e+00 | |
| Burst | 0.129077 | 0.093627 | 1.378620 | 0.168012 | 0.199125 |
| Age | −0.001216 | 0.001361 | −0.893976 | 0.371335 | 0.405403 |
| $N$ | 3654 | | | | |
| Fit RMSD | 0.006111 | | | | |
| $\frac{\text{RMSD}}{\sigma}$ | 0.997784 | | | | |

The complete specification for a one-part fractional regression model fit to predict each day's proportion of created users who shared at least one link to a low-credibility site. Account creation bursts were detected with a probability of false alarm (PFA) of 0.25. Each variable coefficient is provided, along with its robust standard error (SE), $z$-score, raw $p$-value, and corrected $p$-value. The number of data points ($N$), the root-mean-square deviation (RMSD) achieved on the fitted data, and the RMSD divided by the standard deviation of the dependent variable $\left(\frac{\text{RMSD}}{\sigma}\right)$ have also been provided.

Table 23: Regression coefficients when modelling daily proportion of created users sharing low-credibility sites, as computed with medium-sensitivity bursts.

| Variable | Coefficient | SE | $z$ | $p$ | Corrected $p$ |
|---|---|---|---|---|---|
| Intercept | −3.697513 | 0.008771 | −421.577952 | 0e+00 | |
| Burst | 0.174202 | 0.050155 | 3.473262 | 0.000514 | 0.000823 |
| Age | −0.000402 | 0.001367 | −0.294365 | 0.768479 | 0.801903 |
| $N$ | 3654 | | | | |
| Fit RMSD | 0.006075 | | | | |
| $\frac{\text{RMSD}}{\sigma}$ | 0.991884 | | | | |

The complete specification for a one-part fractional regression model fit to predict each day's proportion of created users who shared at least one link to a low-credibility site. Account creation bursts were detected with a probability of false alarm (PFA) of 0.30. Each variable coefficient is provided, along with its robust standard error (SE), $z$-score, raw $p$-value, and corrected $p$-value. The number of data points ($N$), the root-mean-square deviation (RMSD) achieved on the fitted data, and the RMSD divided by the standard deviation of the dependent variable $\left(\frac{\text{RMSD}}{\sigma}\right)$ have also been provided.

Table 24: Regression coefficients when modelling daily proportion of created users sharing low-credibility sites, as computed with high-sensitivity bursts.

| Variable | Coefficient | SE | $z$ | $p$ | Corrected $p$ |
|---|---|---|---|---|---|
| Intercept | −3.705743 | 0.008588 | −431.509285 | 0e+00 | |
| Burst | 0.101629 | 0.014099 | 7.208030 | 6e−13 | 2e−12 |
| Age | −0.001470 | 0.001344 | −1.094311 | 0.273818 | 0.313524 |
| $N$ | 3654 | | | | |
| Fit RMSD | 0.006047 | | | | |
| $\frac{\text{RMSD}}{\sigma}$ | 0.987341 | | | | |

The complete specification for a one-part fractional regression model fit to predict each day's proportion of created users who shared at least one link to a low-credibility site. Account creation bursts were detected with a probability of false alarm (PFA) of 0.35. Each variable coefficient is provided, along with its robust standard error (SE), $z$-score, raw $p$-value, and corrected $p$-value. The number of data points ($N$), the root-mean-square deviation (RMSD) achieved on the fitted data, and the RMSD divided by the standard deviation of the dependent variable $\left(\frac{\text{RMSD}}{\sigma}\right)$ have also been provided.

Table 25: Average marginal effects on daily mean of created users' neighborhood cosine similarities, as computed with low-sensitivity bursts.

| Variable | AME | SE | $z$ | $p$ | Corrected $p$ | $\frac{\text{AME}}{\sigma}$ |
|---|---|---|---|---|---|---|
| Burst | 0.049528 | 0.011279 | 4.391350 | 0.000011 | 0.000023 | 1.377587 |
| Age | −0.003337 | 0.000264 | −12.628227 | 1e−36 | 8e−36 | −0.092823 |
| $N$ | 3654 | | | | | |
| Fit RMSD | 0.033950 | | | | | |
| $\frac{\text{RMSD}}{\sigma}$ | 0.944295 | | | | | |

The average marginal effects (AMEs) for a one-part fractional regression model fit to predict each day's mean neighborhood cosine similarity amongst accounts created on that day. Account creation bursts were detected with a probability of false alarm (PFA) of 0.25. Each variable's AME is provided, along with its robust standard error (SE), $z$-score, raw $p$-value, corrected $p$-value, and AME divided by the standard deviation of all the neighborhood similarities $\left(\frac{\text{AME}}{\sigma}\right)$. The number of data points ($N$), the root-mean-square deviation (RMSD) achieved on the fitted data, and the RMSD divided by the standard deviation of the dependent variable $\left(\frac{\text{RMSD}}{\sigma}\right)$ have also been provided.

Table 26: Average marginal effects on daily mean of created users' neighborhood cosine similarities, as computed with medium-sensitivity bursts.

| Variable | AME | SE | $z$ | $p$ | Corrected $p$ | $\frac{\text{AME}}{\sigma}$ |
|---|---|---|---|---|---|---|
| Burst | 0.040075 | 0.007105 | 5.640436 | 2e−08 | 5e−08 | 1.114664 |
| Age | −0.003253 | 0.000260 | −12.495337 | 8e−36 | 4e−35 | −0.090486 |
| $N$ | 3654 | | | | | |
| Fit RMSD | 0.033749 | | | | | |
| $\frac{\text{RMSD}}{\sigma}$ | 0.938708 | | | | | |

The average marginal effects (AMEs) for a one-part fractional regression model fit to predict each day's mean neighborhood cosine similarity amongst accounts created on that day. Account creation bursts were detected with a probability of false alarm (PFA) of 0.30. Each variable's AME is provided, along with its robust standard error (SE), $z$-score, raw $p$-value, corrected $p$-value, and AME divided by the standard deviation of all the neighborhood similarities $\left(\frac{\text{AME}}{\sigma}\right)$. The number of data points ($N$), the root-mean-square deviation (RMSD) achieved on the fitted data, and the RMSD divided by the standard deviation of the dependent variable $\left(\frac{\text{RMSD}}{\sigma}\right)$ have also been provided.

Table 27: Average marginal effects on daily mean of created users' neighborhood cosine similarities, as computed with high-sensitivity bursts.

| Variable | AME | SE | $z$ | $p$ | Corrected $p$ | $\frac{\text{AME}}{\sigma}$ |
|---|---|---|---|---|---|---|
| Burst | 0.016693 | 0.001801 | 9.270715 | 2e−20 | 9e−20 | 0.464317 |
| Age | −0.003501 | 0.000265 | −13.200758 | 9e−40 | 6e−39 | −0.097367 |
| $N$ | 3654 | | | | | |
| Fit RMSD | 0.033829 | | | | | |
| $\frac{\text{RMSD}}{\sigma}$ | 0.940918 | | | | | |

The average marginal effects (AMEs) for a one-part fractional regression model fit to predict each day's mean neighborhood cosine similarity amongst accounts created on that day. Account creation bursts were detected with a probability of false alarm (PFA) of 0.35. Each variable's AME is provided, along with its robust standard error (SE), $z$-score, raw $p$-value, corrected $p$-value, and AME divided by the standard deviation of all the neighborhood similarities $\left(\frac{\text{AME}}{\sigma}\right)$. The number of data points ($N$), the root-mean-square deviation (RMSD) achieved on the fitted data, and the RMSD divided by the standard deviation of the dependent variable $\left(\frac{\text{RMSD}}{\sigma}\right)$ have also been provided.

Table 28: Average marginal effects on daily mean of created users' clustering coefficients, as computed with low-sensitivity bursts.

| Variable | AME | SE | $z$ | $p$ | Corrected $p$ | $\frac{\text{AME}}{\sigma}$ |
|---|---|---|---|---|---|---|
| Burst | 0.011059 | 0.004318 | 2.560883 | 0.010441 | 0.013545 | 0.758412 |
| Age | −0.003224 | 0.000075 | −42.728435 | 0e+00 | 0e+00 | −0.221092 |
| $N$ | 3654 | | | | | |
| Fit RMSD | 0.010998 | | | | | |
| $\frac{\text{RMSD}}{\sigma}$ | 0.754211 | | | | | |

The average marginal effects (AMEs) for a one-part fractional regression model fit to predict each day's mean clustering coefficient for accounts created on that day. Account creation bursts were detected with a probability of false alarm (PFA) of 0.25. Each variable's AME is provided, along with its robust standard error (SE), $z$-score, raw $p$-value, corrected $p$-value, and AME divided by the standard deviation of all the clustering coefficients $\left(\frac{\text{AME}}{\sigma}\right)$. The number of data points ($N$), the root-mean-square deviation (RMSD) achieved on the fitted data, and the RMSD divided by the standard deviation of the dependent variable $\left(\frac{\text{RMSD}}{\sigma}\right)$ have also been provided.

Table 29: Average marginal effects on daily mean of created users' clustering coefficients, as computed with medium-sensitivity bursts.

| Variable | AME | SE | $z$ | $p$ | Corrected $p$ | $\frac{\text{AME}}{\sigma}$ |
|---|---|---|---|---|---|---|
| Burst | 0.007411 | 0.002340 | 3.167381 | 0.001538 | 0.002344 | 0.508216 |
| Age | −0.003217 | 0.000075 | −42.723493 | 0e+00 | 0e+00 | −0.220609 |
| $N$ | 3654 | | | | | |
| Fit RMSD | 0.011001 | | | | | |
| $\frac{\text{RMSD}}{\sigma}$ | 0.754395 | | | | | |

The average marginal effects (AMEs) for a one-part fractional regression model fit to predict each day's mean clustering coefficient for accounts created on that day. Account creation bursts were detected with a probability of false alarm (PFA) of 0.30. Each variable's AME is provided, along with its robust standard error (SE), $z$-score, raw $p$-value, corrected $p$-value, and AME divided by the standard deviation of all the clustering coefficients $\left(\frac{\text{AME}}{\sigma}\right)$. The number of data points ($N$), the root-mean-square deviation (RMSD) achieved on the fitted data, and the RMSD divided by the standard deviation of the dependent variable $\left(\frac{\text{RMSD}}{\sigma}\right)$ have also been provided.

Table 30: Average marginal effects on daily mean of created users' clustering coefficients, as computed with high-sensitivity bursts.

| Variable | AME | SE | $z$ | $p$ | Corrected $p$ | $\frac{\text{AME}}{\sigma}$ |
|---|---|---|---|---|---|---|
| Burst | 0.000884 | 0.000587 | 1.505647 | 0.132158 | 0.158589 | 0.060591 |
| Age | −0.003270 | 0.000079 | −41.409442 | 0e+00 | 0e+00 | −0.224250 |
| $N$ | 3654 | | | | | |
| Fit RMSD | 0.011069 | | | | | |
| $\frac{\text{RMSD}}{\sigma}$ | 0.759110 | | | | | |

The average marginal effects (AMEs) for a one-part fractional regression model fit to predict each day's mean clustering coefficient for accounts created on that day. Account creation bursts were detected with a probability of false alarm (PFA) of 0.35. Each variable's AME is provided, along with its robust standard error (SE), $z$-score, raw $p$-value, corrected $p$-value, and AME divided by the standard deviation of all the clustering coefficients $\left(\frac{\text{AME}}{\sigma}\right)$. The number of data points ($N$), the root-mean-square deviation (RMSD) achieved on the fitted data, and the RMSD divided by the standard deviation of the dependent variable $\left(\frac{\text{RMSD}}{\sigma}\right)$ have also been provided.

Table 31: Average marginal effects on density of communication between users created on each day, as computed with low-sensitivity bursts.

| Variable | AME | SE | $z$ | $p$ | Corrected $p$ | $\frac{\text{AME}}{\sigma}$ |
|---|---|---|---|---|---|---|
| Burst | 0.000001 | 4e−07 | 2.933526 | 0.003351 | 0.004950 | 0.617066 |
| Age | −5e−08 | 1e−08 | −4.744361 | 0.000002 | 0.000005 | −0.023302 |
| $N$ | 3654 | | | | | |
| Fit RMSD | 0.000002 | | | | | |
| $\frac{\text{RMSD}}{\sigma}$ | 0.993878 | | | | | |

The average marginal effects (AMEs) for a one-part fractional regression model fit to predict the communication density between the users created on each day. Account creation bursts were detected with a probability of false alarm (PFA) of 0.25. Each variable's AME is provided, along with its robust standard error (SE), $z$-score, raw $p$-value, corrected $p$-value, and AME divided by the standard deviation of all the cohort densities $\left(\frac{\text{AME}}{\sigma}\right)$. The number of data points ($N$), the root-mean-square deviation (RMSD) achieved on the fitted data, and the RMSD divided by the standard deviation of the dependent variable $\left(\frac{\text{RMSD}}{\sigma}\right)$ have also been provided.

Table 32: Average marginal effects on density of communication between users created on each day, as computed with medium-sensitivity bursts.

| Variable | AME | SE | $z$ | $p$ | Corrected $p$ | $\frac{\text{AME}}{\sigma}$ |
|---|---|---|---|---|---|---|
| Burst | 9e−07 | 2e−07 | 3.682793 | 0.000231 | 0.000389 | 0.418457 |
| Age | −5e−08 | 1e−08 | −4.583396 | 0.000005 | 0.000010 | −0.022872 |
| $N$ | 3654 | | | | | |
| Fit RMSD | 0.000002 | | | | | |
| $\frac{\text{RMSD}}{\sigma}$ | 0.994084 | | | | | |

The average marginal effects (AMEs) for a one-part fractional regression model fit to predict the communication density between the users created on each day. Account creation bursts were detected with a probability of false alarm (PFA) of 0.30. Each variable's AME is provided, along with its robust standard error (SE), $z$-score, raw $p$-value, corrected $p$-value, and AME divided by the standard deviation of all the cohort densities $\left(\frac{\text{AME}}{\sigma}\right)$. The number of data points ($N$), the root-mean-square deviation (RMSD) achieved on the fitted data, and the RMSD divided by the standard deviation of the dependent variable $\left(\frac{\text{RMSD}}{\sigma}\right)$ have also been provided.

Table 33: Average marginal effects on density of communication between users created on each day, as computed with high-sensitivity bursts.

| Variable | AME | SE | $z$ | $p$ | Corrected $p$ | $\frac{\text{AME}}{\sigma}$ |
|---|---|---|---|---|---|---|
| Burst | 3e−07 | 8e−08 | 4.211855 | 0.000025 | 0.000047 | 0.154952 |
| Age | −5e−08 | 1e−08 | −5.088630 | 4e−07 | 0.000001 | −0.025613 |
| $N$ | 3654 | | | | | |
| Fit RMSD | 0.000002 | | | | | |
| $\frac{\text{RMSD}}{\sigma}$ | 0.995120 | | | | | |

The average marginal effects (AMEs) for a one-part fractional regression model fit to predict the communication density between the users created on each day. Account creation bursts were detected with a probability of false alarm (PFA) of 0.35. Each variable's AME is provided, along with its robust standard error (SE), $z$-score, raw $p$-value, corrected $p$-value, and AME divided by the standard deviation of all the cohort densities $\left(\frac{\text{AME}}{\sigma}\right)$. The number of data points ($N$), the root-mean-square deviation (RMSD) achieved on the fitted data, and the RMSD divided by the standard deviation of the dependent variable $\left(\frac{\text{RMSD}}{\sigma}\right)$ have also been provided.

Table 34: Average marginal effects on daily mean of created users' hashtag cosine similarities, as computed with low-sensitivity bursts.

| Variable | AME | SE | $z$ | $p$ | Corrected $p$ | $\frac{\text{AME}}{\sigma}$ |
|---|---|---|---|---|---|---|
| Burst | 0.009838 | 0.004819 | 2.041599 | 0.041191 | 0.050697 | 2.105514 |
| Age | 0.000028 | 0.000026 | 1.076154 | 0.281858 | 0.318334 | 0.006022 |
| $N$ | 3654 | | | | | |
| Fit RMSD | 0.004534 | | | | | |
| $\frac{\text{RMSD}}{\sigma}$ | 0.970439 | | | | | |

The average marginal effects (AMEs) for a one-part fractional regression model fit to predict each day's mean hashtag cosine similarity amongst accounts created on that day. Account creation bursts were detected with a probability of false alarm (PFA) of 0.25. Each variable's AME is provided, along with its robust standard error (SE), $z$-score, raw $p$-value, corrected $p$-value, and AME divided by the standard deviation of all the hashtag similarities $\left(\frac{\text{AME}}{\sigma}\right)$. The number of data points ($N$), the root-mean-square deviation (RMSD) achieved on the fitted data, and the RMSD divided by the standard deviation of the dependent variable $\left(\frac{\text{RMSD}}{\sigma}\right)$ have also been provided.

Table 35: Average marginal effects on daily mean of created users' hashtag cosine similarities, as computed with medium-sensitivity bursts.

| Variable | AME | SE | $z$ | $p$ | Corrected $p$ | $\frac{\text{AME}}{\sigma}$ |
|---|---|---|---|---|---|---|
| Burst | 0.004546 | 0.002208 | 2.058501 | 0.039542 | 0.049299 | 0.972833 |
| Age | 0.000019 | 0.000030 | 0.633691 | 0.526282 | 0.564321 | 0.004094 |
| $N$ | 3654 | | | | | |
| Fit RMSD | 0.004608 | | | | | |
| $\frac{\text{RMSD}}{\sigma}$ | 0.986266 | | | | | |

The average marginal effects (AMEs) for a one-part fractional regression model fit to predict each day's mean hashtag cosine similarity amongst accounts created on that day. Account creation bursts were detected with a probability of false alarm (PFA) of 0.30. Each variable's AME is provided, along with its robust standard error (SE), $z$-score, raw $p$-value, corrected $p$-value, and AME divided by the standard deviation of all the hashtag similarities $\left(\frac{\text{AME}}{\sigma}\right)$. The number of data points ($N$), the root-mean-square deviation (RMSD) achieved on the fitted data, and the RMSD divided by the standard deviation of the dependent variable $\left(\frac{\text{RMSD}}{\sigma}\right)$ have also been provided.

Table 36: Average marginal effects on daily mean of created users' hashtag cosine similarities, as computed with high-sensitivity bursts.

| Variable | AME | SE | $z$ | $p$ | Corrected $p$ | $\frac{\text{AME}}{\sigma}$ |
|---|---|---|---|---|---|---|
| Burst | 0.000903 | 0.000397 | 2.275951 | 0.022849 | 0.028862 | 0.193214 |
| Age | −0.000012 | 0.000044 | −0.272554 | 0.785196 | 0.801903 | −0.002551 |
| $N$ | 3654 | | | | | |
| Fit RMSD | 0.004658 | | | | | |
| $\frac{\text{RMSD}}{\sigma}$ | 0.996975 | | | | | |

The average marginal effects (AMEs) for a one-part fractional regression model fit to predict each day's mean hashtag cosine similarity amongst accounts created on that day. Account creation bursts were detected with a probability of false alarm (PFA) of 0.35. Each variable's AME is provided, along with its robust standard error (SE), $z$-score, raw $p$-value, corrected $p$-value, and AME divided by the standard deviation of all the hashtag similarities $\left(\frac{\text{AME}}{\sigma}\right)$. The number of data points ($N$), the root-mean-square deviation (RMSD) achieved on the fitted data, and the RMSD divided by the standard deviation of the dependent variable $\left(\frac{\text{RMSD}}{\sigma}\right)$ have also been provided.

Table 37: Average marginal effects on standard deviation of mail-in voting stances of users created on each day, as computed with low-sensitivity bursts.

| Variable | AME | SE | $z$ | $p$ | Corrected $p$ | $\frac{\text{AME}}{\sigma}$ |
|---|---|---|---|---|---|---|
| Burst | −0.016475 | 0.003863 | −4.265149 | 0.000020 | 0.000038 | −2.551159 |
| Age | 0.000166 | 0.000045 | 3.697498 | 0.000218 | 0.000376 | 0.025707 |
| $N$ | 3654 | | | | | |
| Fit RMSD | 0.006127 | | | | | |
| $\frac{\text{RMSD}}{\sigma}$ | 0.948729 | | | | | |

The average marginal effects (AMEs) for a one-part fractional regression model fit to predict each day's standard deviation in mail-in voting stances amongst accounts created on that day. Account creation bursts were detected with a probability of false alarm (PFA) of 0.25. Each variable's AME is provided, along with its robust standard error (SE), $z$-score, raw $p$-value, corrected $p$-value, and AME divided by the standard deviation of all the stance standard deviations $\left(\frac{\text{AME}}{\sigma}\right)$. The number of data points ($N$), the root-mean-square deviation (RMSD) achieved on the fitted data, and the RMSD divided by the standard deviation of the dependent variable $\left(\frac{\text{RMSD}}{\sigma}\right)$ have also been provided.

Table 38: Average marginal effects on standard deviation of mail-in voting stances of users created on each day, as computed with medium-sensitivity bursts.

| Variable | AME | SE | $z$ | $p$ | Corrected $p$ | $\frac{\text{AME}}{\sigma}$ |
|---|---|---|---|---|---|---|
| Burst | −0.009145 | 0.001878 | −4.868486 | 0.000001 | 0.000003 | −1.416130 |
| Age | 0.000170 | 0.000047 | 3.628395 | 0.000285 | 0.000468 | 0.026297 |
| $N$ | 3654 | | | | | |
| Fit RMSD | 0.006227 | | | | | |
| $\frac{\text{RMSD}}{\sigma}$ | 0.964288 | | | | | |

The average marginal effects (AMEs) for a one-part fractional regression model fit to predict each day's standard deviation in mail-in voting stances amongst accounts created on that day. Account creation bursts were detected with a probability of false alarm (PFA) of 0.30. Each variable's AME is provided, along with its robust standard error (SE), $z$-score, raw $p$-value, corrected $p$-value, and AME divided by the standard deviation of all the stance standard deviations $\left(\frac{\text{AME}}{\sigma}\right)$. The number of data points ($N$), the root-mean-square deviation (RMSD) achieved on the fitted data, and the RMSD divided by the standard deviation of the dependent variable $\left(\frac{\text{RMSD}}{\sigma}\right)$ have also been provided.

Table 39: Average marginal effects on standard deviation of mail-in voting stances of users created on each day, as computed with high-sensitivity bursts.

| Variable | AME | SE | $z$ | $p$ | Corrected $p$ | $\frac{\text{AME}}{\sigma}$ |
|---|---|---|---|---|---|---|
| Burst | −0.002221 | 0.000414 | −5.361753 | 8e−08 | 2e−07 | −0.343998 |
| Age | 0.000231 | 0.000054 | 4.319160 | 0.000016 | 0.000031 | 0.035814 |
| $N$ | 3654 | | | | | |
| Fit RMSD | 0.006363 | | | | | |
| $\frac{\text{RMSD}}{\sigma}$ | 0.985239 | | | | | |

The average marginal effects (AMEs) for a one-part fractional regression model fit to predict each day's standard deviation in mail-in voting stances amongst accounts created on that day. Account creation bursts were detected with a probability of false alarm (PFA) of 0.35. Each variable's AME is provided, along with its robust standard error (SE), $z$-score, raw $p$-value, corrected $p$-value, and AME divided by the standard deviation of all the stance standard deviations $\left(\frac{\text{AME}}{\sigma}\right)$. The number of data points ($N$), the root-mean-square deviation (RMSD) achieved on the fitted data, and the RMSD divided by the standard deviation of the dependent variable $\left(\frac{\text{RMSD}}{\sigma}\right)$ have also been provided.

Table 40: Average marginal effects on standard deviation of mask wearing stances of users created on each day, as computed with low-sensitivity bursts.

| Variable | AME | SE | $z$ | $p$ | Corrected $p$ | $\frac{\text{AME}}{\sigma}$ |
|---|---|---|---|---|---|---|
| Burst | −0.010845 | 0.003909 | −2.774219 | 0.005533 | 0.008049 | −1.841324 |
| Age | −0.000396 | 0.000044 | −8.952917 | 3e−19 | 2e−18 | −0.067290 |
| $N$ | 3654 | | | | | |
| Fit RMSD | 0.005662 | | | | | |
| $\frac{\text{RMSD}}{\sigma}$ | 0.961260 | | | | | |

The average marginal effects (AMEs) for a one-part fractional regression model fit to predict each day's standard deviation in mask wearing stances amongst accounts created on that day. Account creation bursts were detected with a probability of false alarm (PFA) of 0.25. Each variable's AME is provided, along with its robust standard error (SE), $z$-score, raw $p$-value, corrected $p$-value, and AME divided by the standard deviation of all the stance standard deviations $\left(\frac{\text{AME}}{\sigma}\right)$. The number of data points ($N$), the root-mean-square deviation (RMSD) achieved on the fitted data, and the RMSD divided by the standard deviation of the dependent variable $\left(\frac{\text{RMSD}}{\sigma}\right)$ have also been provided.

Table 41: Average marginal effects on standard deviation of mask wearing stances of users created on each day, as computed with medium-sensitivity bursts.

| Variable | AME | SE | $z$ | $p$ | Corrected $p$ | $\frac{\text{AME}}{\sigma}$ |
|---|---|---|---|---|---|---|
| Burst | −0.004902 | 0.001846 | −2.655001 | 0.007931 | 0.010877 | −0.832364 |
| Age | −0.000385 | 0.000046 | −8.322314 | 9e−17 | 3e−16 | −0.065429 |
| $N$ | 3654 | | | | | |
| Fit RMSD | 0.005741 | | | | | |
| $\frac{\text{RMSD}}{\sigma}$ | 0.974743 | | | | | |

The average marginal effects (AMEs) for a one-part fractional regression model fit to predict each day's standard deviation in mask wearing stances amongst accounts created on that day. Account creation bursts were detected with a probability of false alarm (PFA) of 0.30. Each variable's AME is provided, along with its robust standard error (SE), $z$-score, raw $p$-value, corrected $p$-value, and AME divided by the standard deviation of all the stance standard deviations $\left(\frac{\text{AME}}{\sigma}\right)$. The number of data points ($N$), the root-mean-square deviation (RMSD) achieved on the fitted data, and the RMSD divided by the standard deviation of the dependent variable $\left(\frac{\text{RMSD}}{\sigma}\right)$ have also been provided.

Table 42: Average marginal effects on standard deviation of mask wearing stances of users created on each day, as computed with high-sensitivity bursts.

| Variable | AME | SE | $z$ | $p$ | Corrected $p$ | $\frac{\text{AME}}{\sigma}$ |
|---|---|---|---|---|---|---|
| Burst | −0.001054 | 0.000387 | −2.727530 | 0.006381 | 0.008965 | −0.179022 |
| Age | −0.000352 | 0.000052 | −6.712755 | 2e−11 | 7e−11 | −0.059749 |
| $N$ | 3654 | | | | | |
| Fit RMSD | 0.005788 | | | | | |
| $\frac{\text{RMSD}}{\sigma}$ | 0.982712 | | | | | |

The average marginal effects (AMEs) for a one-part fractional regression model fit to predict each day's standard deviation in mask wearing stances amongst accounts created on that day. Account creation bursts were detected with a probability of false alarm (PFA) of 0.35. Each variable's AME is provided, along with its robust standard error (SE), $z$-score, raw $p$-value, corrected $p$-value, and AME divided by the standard deviation of all the stance standard deviations $\left(\frac{\text{AME}}{\sigma}\right)$. The number of data points ($N$), the root-mean-square deviation (RMSD) achieved on the fitted data, and the RMSD divided by the standard deviation of the dependent variable $\left(\frac{\text{RMSD}}{\sigma}\right)$ have also been provided.

Table 43: Average marginal effects on daily proportion of users created with bot scores $\geq 70\%$, as computed with low-sensitivity bursts.

| Variable | AME | SE | $z$ | $p$ | Corrected $p$ | $\frac{\text{AME}}{\sigma}$ |
|---|---|---|---|---|---|---|
| Burst | 0.021641 | 0.004516 | 4.791736 | 0.000002 | 0.000004 | 0.413176 |
| Age | −0.014160 | 0.000260 | −54.419922 | 0e+00 | 0e+00 | −0.270340 |
| $N$ | 3654 | | | | | |
| Fit RMSD | 0.031500 | | | | | |
| $\frac{\text{RMSD}}{\sigma}$ | 0.601400 | | | | | |

The average marginal effects (AMEs) for a one-part fractional regression model fit to predict each day's proportion of created users with bot scores $\geq 70\%$. Account creation bursts were detected with a probability of false alarm (PFA) of 0.25. Each variable's AME is provided, along with its robust standard error (SE), $z$-score, raw $p$-value, corrected $p$-value, and AME divided by the standard deviation of all the bot proportions $\left(\frac{\text{AME}}{\sigma}\right)$. The number of data points ($N$), the root-mean-square deviation (RMSD) achieved on the fitted data, and the RMSD divided by the standard deviation of the dependent variable $\left(\frac{\text{RMSD}}{\sigma}\right)$ have also been provided.

Table 44: Average marginal effects on daily proportion of users created with bot scores $\geq 70\%$, as computed with medium-sensitivity bursts.

| Variable | AME | SE | $z$ | $p$ | Corrected $p$ | $\frac{\text{AME}}{\sigma}$ |
|---|---|---|---|---|---|---|
| Burst | 0.016251 | 0.003538 | 4.593828 | 0.000004 | 0.000009 | 0.310274 |
| Age | −0.014131 | 0.000259 | −54.484879 | 0e+00 | 0e+00 | −0.269789 |
| $N$ | 3654 | | | | | |
| Fit RMSD | 0.031452 | | | | | |
| $\frac{\text{RMSD}}{\sigma}$ | 0.600500 | | | | | |

The average marginal effects (AMEs) for a one-part fractional regression model fit to predict each day's proportion of created users with bot scores $\geq 70\%$. Account creation bursts were detected with a probability of false alarm (PFA) of 0.30. Each variable's AME is provided, along with its robust standard error (SE), $z$-score, raw $p$-value, corrected $p$-value, and AME divided by the standard deviation of all the bot proportions $\left(\frac{\text{AME}}{\sigma}\right)$. The number of data points ($N$), the root-mean-square deviation (RMSD) achieved on the fitted data, and the RMSD divided by the standard deviation of the dependent variable $\left(\frac{\text{RMSD}}{\sigma}\right)$ have also been provided.

Table 45: Average marginal effects on daily proportion of users created with bot scores $\geq 70\%$, as computed with high-sensitivity bursts.

| Variable | AME | SE | $z$ | $p$ | Corrected $p$ | $\frac{\text{AME}}{\sigma}$ |
|---|---|---|---|---|---|---|
| Burst | 0.000332 | 0.001431 | 0.232268 | 0.816330 | 0.816330 | 0.006344 |
| Age | −0.014267 | 0.000257 | −55.428994 | 0e+00 | 0e+00 | −0.272399 |
| $N$ | 3654 | | | | | |
| Fit RMSD | 0.031669 | | | | | |
| $\frac{\text{RMSD}}{\sigma}$ | 0.604641 | | | | | |

The average marginal effects (AMEs) for a one-part fractional regression model fit to predict each day's proportion of created users with bot scores $\geq 70\%$. Account creation bursts were detected with a probability of false alarm (PFA) of 0.35. Each variable's AME is provided, along with its robust standard error (SE), $z$-score, raw $p$-value, corrected $p$-value, and AME divided by the standard deviation of all the bot proportions $\left(\frac{\text{AME}}{\sigma}\right)$. The number of data points ($N$), the root-mean-square deviation (RMSD) achieved on the fitted data, and the RMSD divided by the standard deviation of the dependent variable $\left(\frac{\text{RMSD}}{\sigma}\right)$ have also been provided.

Table 46: Average marginal effects on daily proportion of created users who shared links to low-credibility sites, as computed with low-sensitivity bursts.

| Variable | AME | SE | $z$ | $p$ | Corrected $p$ | $\frac{\text{AME}}{\sigma}$ |
|---|---|---|---|---|---|---|
| Burst | 0.003247 | 0.002501 | 1.298009 | 0.194284 | 0.227455 | 0.530104 |
| Age | −0.000029 | 0.000032 | −0.893444 | 0.371619 | 0.405403 | −0.004705 |
| $N$ | 3654 | | | | | |
| Fit RMSD | 0.006111 | | | | | |
| $\frac{\text{RMSD}}{\sigma}$ | 0.997784 | | | | | |

The average marginal effects (AMEs) for a one-part fractional regression model fit to predict each day's proportion of created users who shared links to at least one low-credibility site. Account creation bursts were detected with a probability of false alarm (PFA) of 0.25. Each variable's AME is provided, along with its robust standard error (SE), $z$-score, raw $p$-value, corrected $p$-value, and AME divided by the standard deviation of all the bot proportions $\left(\frac{\text{AME}}{\sigma}\right)$. The number of data points ($N$), the root-mean-square deviation (RMSD) achieved on the fitted data, and the RMSD divided by the standard deviation of the dependent variable $\left(\frac{\text{RMSD}}{\sigma}\right)$ have also been provided.

Table 47: Average marginal effects on daily proportion of created users who shared links to low-credibility sites, as computed with medium-sensitivity bursts.

| Variable | AME | SE | $z$ | $p$ | Corrected $p$ | $\frac{\text{AME}}{\sigma}$ |
|---|---|---|---|---|---|---|
| Burst | 0.004462 | 0.001392 | 3.205733 | 0.001347 | 0.002120 | 0.728565 |
| Age | −0.000010 | 0.000032 | −0.294304 | 0.768526 | 0.801903 | −0.001557 |
| $N$ | 3654 | | | | | |
| Fit RMSD | 0.006075 | | | | | |
| $\frac{\text{RMSD}}{\sigma}$ | 0.991884 | | | | | |

The average marginal effects (AMEs) for a one-part fractional regression model fit to predict each day's proportion of created users who shared links to at least one low-credibility site. Account creation bursts were detected with a probability of false alarm (PFA) of 0.30. Each variable's AME is provided, along with its robust standard error (SE), $z$-score, raw $p$-value, corrected $p$-value, and AME divided by the standard deviation of all the bot proportions $\left(\frac{\text{AME}}{\sigma}\right)$. The number of data points ($N$), the root-mean-square deviation (RMSD) achieved on the fitted data, and the RMSD divided by the standard deviation of the dependent variable $\left(\frac{\text{RMSD}}{\sigma}\right)$ have also been provided.

Table 48: Average marginal effects on daily proportion of created users who shared links to low-credibility sites, as computed with high-sensitivity bursts.

| Variable | AME | SE | $z$ | $p$ | Corrected $p$ | $\frac{\text{AME}}{\sigma}$ |
|---|---|---|---|---|---|---|
| Burst | 0.002481 | 0.000358 | 6.922871 | 4e−12 | 2e−11 | 0.405123 |
| Age | −0.000035 | 0.000032 | −1.093137 | 0.274334 | 0.313524 | −0.005686 |
| $N$ | 3654 | | | | | |
| Fit RMSD | 0.006047 | | | | | |
| $\frac{\text{RMSD}}{\sigma}$ | 0.987341 | | | | | |

The average marginal effects (AMEs) for a one-part fractional regression model fit to predict each day's proportion of created users who shared links to at least one low-credibility site. Account creation bursts were detected with a probability of false alarm (PFA) of 0.35. Each variable's AME is provided, along with its robust standard error (SE), $z$-score, raw $p$-value, corrected $p$-value, and AME divided by the standard deviation of all the bot proportions $\left(\frac{\text{AME}}{\sigma}\right)$. The number of data points ($N$), the root-mean-square deviation (RMSD) achieved on the fitted data, and the RMSD divided by the standard deviation of the dependent variable $\left(\frac{\text{RMSD}}{\sigma}\right)$ have also been provided.
