# Supplementary material for: Investigating coordinated account creation using burst detection and network analysis
Source: J Big Data. 2023 Feb 10;10(1):20. doi: 10.1186/s40537-023-00695-7 (PMC9913025; doi:10.1186/s40537-023-00695-7)
Supplement: Supplementary file 2 — Additional file 2. Additional methods. [file 40537_2023_695_MOESM2_ESM.pdf]

Supplementary methods for "Investigating coordinated account creation using burst detection and network analysis"

Daniele Bellutta & Kathleen M. Carley

Terms Tracked in Data Collection

The Twitter streaming API was used to gather election-related tweets during a period of about 14 months. The specific terms being tracked in this data collection were changed over the course of that period in order to remain relevant to the broad themes of election-related discussions. The following sections list the specific hashtags, users, and other terms for which data were collected, along with the time periods during which they were used in our data collection.

1 December 2019 to 16 August 2020

#election2020, #2020_presidential_election, #maga2020, #flipitblue, #keepitblue, #yeswecan, #yang2020, #JoeBiden, #BernieSanders, #ElizabethWarren, #PeteButtigieg, #FeelTheBern, #democrats, #republicans, #Bloomberg2020, #Booker, @TulsiGabbard, @GovBillWeld, @AndrewYang, @TomSteyer, @JohnDelaney, @ewarren, @WalshFreedom, @PeteButtigieg, @BernieSanders, @Devalpatrick, @MichaelBennet, @AmyKlobuchar, @TulsiGabbard, @marwilliamson, @JulianCastro, @MikeBloomberg, @CoryBooker, @JoeBiden, @realDonaldTrump

16 August 2020 to 15 January 2021

#USPS, #VoteByMail, #SaveTheUSPS, #voterfraud, #BlackLivesMatter, #BLM, #reopen, #reopenamerica, #IranSanctions, #QAnon, #WWG1WGA, "natural born", @USPS, #election2020, #presidentialelection, #democrats, #republicans, #JoeBiden, #BidenHarris2020, #Biden, #MAGA, #KAG, @JoeBiden, @realDonaldTrump, @POTUS, @Mike_Pence, @VP, @KamalaHarris, @SenKamalaHarris, @CoryGardner, @SenCoryGardner, @Hickenlooper, @Perduesenate, @sendavidperdue, @ossoff, @joniernst, @SenJoniErnst, @GreenfieldIowa, @SenSusanCollins, @SenatorCollins, @SaraGideon, @SteveDaines, @stevebullockmt, @GovernorBullock, @ThomTillis, @SenThomTillis, @CalforNC

15 January 2021 to 17 February 2021

@JoeBiden, @realDonaldTrump, @POTUS, @Mike_Pence, @VP, @KamalaHarris, @SenKamalaHarris, @USPS, inauguration, #Inauguration, #InaugurationDay, #Capitol, #USCapitol, #USCapital, #NationalMall, #Jan20, #election2020, #presidentialelection, #democrats, #republicans, #JoeBiden, #Biden, #MAGA, #KAG, #Trump, #USPS, #VoteByMail, #SaveTheUSPS, #voterfraud, #BlackLivesMatter, #BLM, #reopen, #reopenamerica, #IranSanctions, #QAnon, #WWG1WGA, "natural born"

Hashtags Used to Initialize Stance Detection

Since the stance detection algorithm required an initial set of hashtags labeled by stance, we manually labeled sets of popular hashtags in favor of and against mail-in voting and mask wearing. The following sections list these hashtags by issue and stance.

Hashtags in Favor of Mail-in Voting

#savetheusps, #votebymail, #votebymail2020, #saveusps, #uspsisessential, #dontmesswithusps, #votebymailearly, #mailthevotega, #protectuspswhistleblowers, #usps2020vote, #protectusps, #votebymail4bernie, #usps_is_essential, #fundusps, #wewillvotebymail, #mailthevotefl, #protecttheusps, #votebluebymail2020, #savetheusps2020, #supportusps, #votebymailnow, #fundtheusps, #uspsismylifeline, #saveourusps, #uspsforever, #voteabsenteemailblue, #dontmesswiththeusps, #votebluebymail, #restoreusps, #votebymailforall

Hashtags Against Mail-in Voting

#mailinvoterfraud, #mailinballotfraud, #nomailinvoting, #exposeusps, #nomailinballots, #novotebymail, #mailinvotingfraud, #demvotebymailscam, #uspslies, #mailvoterfraud, #hellnotovotebymail, #mailinvotefraud, #stopvotebymail, #ballotbymailfraud, #mailinballotsfraud, #defundusps, #nomailinvote, #votebymailfraud, #defundtheusps, #votebymailisfraud, #exposetheusps, #nomailinvotes, #fraudulentmailinballots, #mailinballotswouldbeatotaldisater, #stopmailinvoting, #fakemailvotes, #nomailvoting, #dontmailyourballot, #uspssucks, #voterfraudbymail

Hashtags in Favor of Mask Wearing

#wearamask, #wearadamnmask, #maskup, #maskssavelives, #wearamasksavealife, #masks4all, #realmenwearmasks, #maskupamerica, #wearthedamnmask, #masksforall, #wearthemask, #mandatemasks, #wearyourmask, #makefacemaskmandatory, #wearamaskplease

Hashtags Against Mask Wearing

#masksoffamerica, #masksoff, #unmaskamerica, #masksdontwork, #nomaskmandates, #nomoremasks, #maskoff, #nomaskonme, #burnyourmask, #masksdonotwork, #takeoffthemask, #maskhoax, #burnthemask, #sheepwearmasks, #trashthemask

Low-Credibility Web Domains

The following Web domains were used to determine which users authored tweets containing links to low-credibility sites.

100percentfedup.com
10news.one
12minutos.com
16wmpo.com
20minutenews.com
247newsmedia.com
24aktuelles.com
24online.news
24usainfo.com
24wpn.com
24x365live.com
911truth.org
a-news24.com
aattp.org
abcbusinessnews.com
abcnews-us.com
abcnews.com.co
abovetopsecret.com
acpeds.org
actionnews3.com
activistmommy.com
activistpost.com

actualite.co
actualites.co
adflegal.org
adobochronicles.com
afa.net
ageofautism.com
ageofshitlords.com
alabamaobserver.com
aldipest.com
alertchild.com
allenwestrepublic.com
allnewspipeline.com
alphanewsmn.com
alternativenews.com
althealthworks.com
altright.com
alynews.com
amazingnews.net
americafans.com
americanflare.com
americanflavor.news
americanfreepress.net
americanjournalreview.com
americanlookout.com
americannews.com
americanpatriotdaily.com
americanpeoplenetwork.com
americanpoliticnews.co
americanpresident.co
americanprides.com
americanprides.org
americanprinciplesproject.org
americansfortruth.com
americanthinker.com
americantoday.news
americantoday.us
americantruthseekers.com
americanupdater.com
americasfreedomfighters.com
americasnewest.com
americatalks.com
amposts.com
amren.com
amtvmedia.com
anews-24.com
anews24.org
angrypatriotmovement.com
anonhq.com
anonjekloy.tk
answersingenesis.org
antinews.com
antinewsnetwork.com
areyouasleep.com
armyusanews.com
asamericanasapplepie.org
asheepnomore.net

assassinationscience.com
associatedmediacoverage.com
aurora-news.us
australiannationalreview.com
autisminvestigated.com
avoiceformen.com
awarenessact.com
backroombuzz.com
badcriminals.com
baldwinpost.com
baltimoregazette.com
barenakedislam.com
battypost.com
bb4sp.com
beaware.one
beforeitsnews.com
bients.com
bigbluevision.org
biggovernment.news
bigleaguepolitics.com
bignuggetnews.com
billionbibles.org
bipartisanreport.com
bizstandardnews.com
blackeyepolitics.com
blackgenocide.org
blackpigeonspeaks.com
blingnews.com
bloodandsoil.org
blueinformer.com
bluetribune.com
bluevision.news
bluevisionpost.com
bluntforcetruth.com
borderherald.com
borderlandalternativemedia.com
bostonleader.com
breaking911.com
breakingnews247.net
breakingnews365.net
breakingnewsblast.com
breakingtop.world
breitbart.com
britainfirst.tv
burrardstreetjournal.com
butthatsnoneofmybusiness.com
buzzfeedusa.com
canadafreepress.com
cap-news.com
carm.org
cartelpress.com
cartelreport.com
cbsnews.com.co
cei.org
celebtricity.com
cfact.org

channel16news.com
channel17news.com
channel18news.com
channel22news.com
channel23news.com
channel24news.com
channel28news.com
channel34news.com
channel45news.com
channel5000.com
checkpointasia.net
chicagodaily.pro
choiceandtruth.com
christianaction.org
christianscience.com
christiantimesnewspaper.com
christiantoday.info
cityworldnews.com
civictribune.com
clancyreport.com
clarionproject.org
clashdaily.com
clear-politics.com
climatechangedispatch.com
climatescienceinternational.org
climatism.blog
cloverchronicle.com
cnewsgo.com
cnn-business-news.ga
cnn-globalnews.com
cnnews3.com
cnsnews.com
coasttocoastam.com
coffeebreakforyou.com
collective-evolution.com
collectivelyconscious.net
com.de
concernedwomen.org
consciouslyenlightened.com
conservative101.com
conservativearmy88.com
conservativebuzz.com
conservativebyte.com
conservativedailypost.com
conservativefighters.com
conservativeflashnews.com
conservativefreepress.com
conservativeinfocorner.com
conservativeinfocorner.us
conservativeinsider.co
conservativemedia.com
conservativepaper.com
conservativepoliticus.com
conservativepost.com
conservativespirit.com
conservativestudio.com

conservativeview.info
consmovement.com
consnation.com
conspatriot.com
conspiracydailyupdate.com
conspiracyinquirer.com
conspiracyplanet.com
cooltobeconservative.com
corbettreport.com
cosmicintelligenceagency.com
countdowntozerotime.com
creation.com
csglobe.com
curiousmindmagazine.com
daily-sun.com
dailybuzzlive.com
dailycurrant.com
dailyfeed.news
dailyfinesser.com
dailyheadlines.net
dailyhealthpost.com
dailyinfobox.com
dailyinsidernews.com
dailymail.co.uk
dailynews10.com
dailynews11.com
dailynews3.com
dailynews33.com
dailynews5.com
dailynewsposts.info
dailynotify.com
dailyoccupation.com
dailypresser.com
dailysidnews.com
dailystormer.name
dailysurge.com
dailythings.world
dailyusaupdate.com
dailyworldinformation.com
damnleaks.com
damreports.com
dangerandplay.com
darkjournalist.com
dataasylum.com
davidicke.com
dcclothesline.com
dcdirtylaundry.com
debunkingskeptics.com
defendevropa.org
defenseusa.club
defiantamerica.com
defund.com
democraticmoms.com
democraticreview.com
democraticunderground.com
denverguardian.com

denverinquirer.com
departed.co
departedme.com
departedmedia.com
deplorablekel.com
deplorablemovement2020.news
digifection.com
dineal.com
disclose.tv
discoverthenetworks.org
discovery.org
diyhours.net
donaldtrumpnews.co
donaldtrumppotus45.com
downtrend.com
drainingtheswamp.info
drugsofficial.com
eaglerising.com
earthpulse.com
educate-yourself.org
embols.com
empireherald.com
empirenews.net
empiresports.co
en-volve.com
endingthefed.com
endthefed.org
endtimeheadlines.org
enhlive.com
escapeallthesethings.com
everydaybreakingnews.com
everynewshere.com
evolutionnews.org
ewao.com
explainlife.com
exposinggovernment.com
fairus.org
famousviralstories.com
fanzinger.com
fbnewscycle.com
federalistnation.com
federalisttribune.com
fedsalert.com
fellowshipoftheminds.com
flashinfo.org
flashnewscorner.com
floridasunpost.com
focusnews.info
focusonthefamily.com
folksvideo.com
foreverconscious.com
forfreedomworld.com
fox-news24.com
france24-tv.com
frc.org
freakoutnation.com

freddymag.com
freedomadvocates.org
freedomcrossroads.us
freedomdaily.com
freedomlibertynews.com
freedomproject.com
freedomsfinalstand.com
freeinfomedia.com
freepatriotpost.com
freshdailyreport.com
fromthetrenchesworldreport.com
frontpagemag.com
fury.news
gellerreport.com
geoengineeringwatch.org
getcancerremedies.com
girlsjustwannahaveguns.com
givemeliberty01.com
globalassociatednews.com
globalinfotoday.com
globalpoliticsnow.com
globalresearch.ca
globalrevolutionnetwork.com
globalskywatch.com
globemagazine.com
godandscience.org
godlikeproductions.com
goneleft.com
goodgopher.com
gotnews.com
gotquestions.org
govtslaves.com
greatamericanrepublic.com
greenvillegazette.com
guerilla.news
guerillanews.com
gulagbound.com
gummypost.com
haarp.net
halturnershow.com
hangthebankers.com
hawarnews.com
headlinebrief.com
healingoracle.ch
healthimpactnews.com
healthnutnews.com
healthy-holistic-living.com
healthy-vibes.com
healthyworldhouse.com
heartland.org
heaviermetal.net
hoggwatch.com
holylandnutrition.com
hotglobalnews.com
houstonchronicle-tv.com
hsionline.com

humansarefree.com
huzlers.com
iceagenow.info
icr.org
identityevropa.com
ifyouonlynews.com
ihatethemedia.com
ihavethetruth.com
ihealthtube.com
ihr.org
illicitinfo.com
illuminati-news.com
illuminatiwatcher.com
ilovemyfreedom.org
imowired.com
indigenous.club
infiniteunknown.net
infogalactic.com
informationliberation.com
informedowners.com
infowars.com
intellihub.com
interestingdailynews.com
intrendtoday.com
iowaclimate.org
ipatriot.com
isthatlegit.com
itaglive.com
jesus-is-savior.com
jesusdaily.com
jewsnews.co.il
jewwatch.com
joeforamerica.com
jokerviral.com
jonesreport.com
jookos.com
judicialwatch.org
junkscience.com
kata33.com
kbc14.com
kcna.kp
kcst7.com
kf13.com
klponews.com
km8news.com
kmt11.com
knightstemplarinternational.com
knightstemplarorder.com
knowledgeoftoday.org
knp7.com
konkonsagh.biz
krb7.com
krbcnews.com
kspm33.com
kty24news.com
kupr7.com

ky12news.com
ky6news.com
kypo6.com
ladbible.com
ladylibertysnews.com
landrypost.com
larouchepac.com
lastdeplorables.com
lastresistance.com
learnprogress.org
learntherisk.org
leftexposed.org
lewrockwell.com
liberalplug.com
liberalsociety.com
liberty-courier.com
libertyalliance.com
libertybrief.com
libertyisviral.com
libertyvideos.org
libertywritersnews.com
local31news.com
localnews33.com
londonwebnews.com
loonwatch.com
lopezreport.com
madamericannetwork.com
madworldnews.com
maga2020.us
magavoter.com
mainerepublicemailalert.com
majorthoughts.com
martinlutherking.org
maywoodpost.com
mbganews.com
mbynews.com
mckenziepost.com
mediamass.net
mediazone.news
medicine.news
meforum.org
mercola.com
metapedia.org
metropolitanworlds.com
mississippiherald.com
mixi.media
molonlabemedia.com
moonbattery.com
morningledger.com
morningnewsusa.com
movingleft.com
mrcblog.com
mrnewswatch.com
msfanpage.link
msnbc.website
mygoogle.ca

mynewsguru.com
myrightamerica.com
natall.com
nationaleconomicseditorial.com
nationalenquirer.com
nationalinsiderpolitics.com
nationalpolicy.institute
nationalreport.net
nationalvanguard.org
nativestuff.us
naturalcuresnotmedicine.info
naturalmedicine.news
naturalnews.com
naturalnewsblogs.com
nbc.com.co
ncscooper.com
needtoknow.news
neonnettle.com
nephef.com
neverrepublican.com
nevo.news
newcenturytimes.com
newnation.org
newobserveronline.com
newpoliticstoday.com
news14kgpn.com
news14now.com
news4ktla.com
newsandstoriesfromusa.com
newsbiscuit.com
newsbreakhere.com
newsbreakingspipe.com
newsbreakshere.com
newsbuzzdaily.com
newsbysquad.com
newschicken.com
newsconservative.com
newsdaily10.com
newsdaily12.com
newsdaily27.com
newsexaminer.net
newsfeedhunter.com
newsfeedobserver.com
newsformetoday.com
newshubs.info
newsinsideout.com
newsleak.co
newslo.com
newsner.com
newsnow17.com
newsoftrump.com
newsphd.com
newspunch.com
newstarget.com
newsuptoday.com
newswars.com

newswatch33.com
newswithviews.com
newzmagazine.com
nmws.us
nnettle.com
nodisinfo.com
noscomunicamos.com
notallowedto.com
noticias365.info
notrickszone.com
now8news.com
nowtheendbegins.com
npiamerica.org
nvic.org
nydailynews-tv.com
nymeta.co
oathkeepers.org
observeronline.news
occupydemocrats.com
onepoliticalplaza.com
onlineconservativepress.com
openmagazines.com
oreillypost.com
other98.com
oureyeonislam.com
ourhealthguides.com
ourlandofthefree.com
oye.news
pacificpundit.com
pakalertpress.com
pamelageller.com
patribotics.blog
patriotbeacon.com
patriotcrier.com
patriotfires.com
patriothangout.com
patrioticexpress.com
patrioticviralnews.com
patriotsvoice.info
patriotupdate.com
patriotusa.website
persecutes.com
personalliberty.com
plymouthpatriotpost.com
politicalblindspot.com
politicalears.com
politicalflare.com
politicalinbox.com
politicalmayhem.news
politicalsitenews.com
politicoinfo.com
politicops.com
politicot.com
politics.com
politicslive.net
politicspaper.com

politicsusanews.com
politifact.news
powderedwigsociety.com
powerdaily.us
powerofnative.com
prageru.com
prepareforchange.net
president45donaldtrump.com
pressunion.org
prideof-america.org
principia-scientific.org
prisonplanet.com
prntly.com
proamericanews.com
profam.org
progressivestoday.com
projectpurge.com
prophecynewswatch.com
prophecytoday.com
proud-patriots.com
proudcons.com
proudleader.com
puppetstringnews.com
qanon.pub
qualitysharing.com
rapturenewsnetwork.com
react365.com
readconservatives.news
realconservativesunite.com
realfarmacy.com
realjewnews.com
realnews24.com
realnewsrightnow.com
realtimepolitics.com
rearfront.com
redcountry.us
redice.tv
redinfo.us
redpeople.us
redpolitics.us
redrocktribune.com
redstatejournalist.com
redstatepundit.com
redstatewatcher.com
redstatewave.com
redwhiteandright.com
reedcooper.net
reflectionofmind.org
religionmind.com
remnantnewspaper.com
renewamerica.com
rense.com
researchantisemitism.ca
returnofkings.com
returntonow.net
revolutionradio.org

revolutions2040.com
rickwells.us
rightalerts.com
rightjournalist.com
rightsidenews.com
rightwing.news
rightwingnews.com
rilenews.com
rinf.com
rodong.rep.kp
rogue-nation3.com
rrtribune.com
rt.com
rumorjournal.com
ruthinstitute.org
rwnofficial.com
sanevax.org
satiratribune.com
savethemales.ca
scrapetv.com
sgtreport.com
sharednewsreport.com
shariawatch.org.uk
sheepkillers.com
shoebat.com
shtfplan.com
sickchirpse.com
skeptiko.com
smhwtfnews.com
smobserved.com
smoloko.com
snoopack.com
socialeverythings.com
southernconservativeextra.com
southjerseymechanic.com
speakgov.com
speisa.com
spinzon.com
sputniknews.com
squawker.org
staresattheworld.com
statefort.com
statenation.co
stateofthenation.co
stateofthenation2012.com
states-tv.com
stgeorgegazette.com
stonecoldtruth.com
stormcloudsgathering.com
stormfront.org
straightstoned.com
strategic-culture.org
stuppid.com
subjectpolitics.com
success-street.com
summit.news

sundayinquirer.com
supremepatriot.com
surenews.com
survivalblog.com
sustainablepulse.com
svijmedia.com
swampdrain.com
takimag.com
tashnews.com
tdnewswire.com
tdtalliance.com
teaparty.org
teddystick.com
telegraphsun.com
teoinfo.com
tfp.org
the-global-news.com
the-insider.co
the-postillon.com
theamericanmirror.com
theavocadonews.com
theaware.net
thebostontribune.com
thebreakingnews.co
thebreakingnews.today
thecommonsenseshow.com
thecontroversialfiles.net
thedailybell.com
thedailyconspiracy.com
thedailysheeple.com
thedcgazette.com
thedeplorablesociety.com
theduran.com
theeventchronicle.com
theexaminer.site
thefederalistpapers.org
theforbiddenknowledge.com
thefreepatriot.org
thefreethoughtproject.com
thefrt.com
thegatewaypundit.com
thegoldwater.com
thegopwatchdog.com
thehealthconsciousness.com
thehornnews.com
theinternationalreporter.org
theinternetpost.net
thelastgreatstand.com
thelastlineofdefense.org
thelibertybeacon.com
themiamigazette.com
themillenniumreport.com
themindunleashed.com
themoralofthestory.us
thenationalpatriot.com
thenationalsun.com

thenet24h.com
thenewsnerd.com
thenewyorkevening.com
thenochill.com
thepeoplestruth.com
thepoliticalinsider.com
thepoliticaltribune.com
thepolitics.online
thepremiumnews.com
thepublicdiscourse.com
theracketreport.com
theredelephants.com
thereligionofpeace.com
thereporterz.com
therightists.com
therightstuff.biz
therundownlive.com
theseattletribune.com
thetrumpmedia.com
thetruthdivision.com
thetruthseeker.co.uk
theusa-news.com
theusaconservative.com
theuspatriot.com
thevalleyreport.com
theviralpatriot.com
thewashingtonpress.com
thewashingtonpundit.com
theworldupdate.com
thinkamericana.com
thinkingmomsrevolution.com
thirdestatenewsgroup.com
thoughtcrimeradio.net
thrivemovement.com
times.com.mx
timespolice.com
tmn.today
tmzbreaking.com
tmzbusiness.com
tmzcomedy.com
tmzhiphop.com
tmzuncut.com
tmzurban.com
tmzworldnews.com
tmzworldstarnews.com
todaydispatch.com
topinfopost.com
toprightnews.com
toptopic.club
tpusa.com
triggerreset.net
trueactivist.com
trueamericans.me
truebluescoop.com
truepundit.com
truetrumpers.com

trueviralnews.com
trump.news
trumpers.online
trumpservativenews.info
trunews.com
truthandaction.org
truthbroadcastnetwork.com
truthchannelpolitics.com
truthcommand.com
truthexam.com
truthfeednews.com
truthinmedia.com
truthkings.com
truthmonitor.com
truthrevolt.org
truthuncensored.net
turningpoint.news
twisted.news
unclesamsmisguidedchildren.com
undergroundhealth.com
undergroundjournalist.org
undergroundnewsreport.com
understandingthethreat.com
uniquewebmagazine.com
unitedmediapublishing.com
universepolitics.com
unz.com
us24news.com
usa-conservative.com
usa-radio.com
usa-television.com
usa360-tv.com
usaconservativereport.com
usadailyinfo.com
usadailypost.us
usadailyreview.com
usadailythings24.com
usadailytime.com
usadosenews.com
usafirstinformation.com
usafortrumponline.com
usahitman.com
usainfront.com
usanetwork.info
usanews4u.us
usanewsflash.com
usanewshome.com
usanewstoday.com
usanewstoday.org
usapolitics24hrs.com
usapoliticsnow.com
usapoliticstoday.com
usapoliticszone.com
usapubliclife.com
usareally.com
usasnich.com

usasupreme.com
usatodaynews.me
usatodaypolitics.com
usaviralnewstoday.com
usawatchdog.com
usaworldbox.com
uschronicle.com
ushealthyadvisor.com
ushealthylife.com
usherald.com
usinfonews.com
usjournalreview.com
uslibertywire.club
uslibertywire.com
uspoliticsinfo.com
uspoln.com
uspostman.com
ustruthwire.com
vaccines.news
vaccinesrevealed.com
vactruth.com
vaxxter.com
vdare.com
vesselnews.io
veteransfordonaldtrump.com
vidmax.com
vigilantcitizen.com
viralactions.com
viralcocaine.com
viralcords.com
viraldevil.com
viralliberty.com
viralmugshot.com
viralnewsnetwork.net
viralpropaganda.com
viralspeech.com
viralstuppid.com
voiceofamericatv.com
voiceofeurope.com
voxtribune.com
vrevealed.com
wakeup-world.com
wakingtimes.com
walkwithher.com
wallbuilders.com
washingtonevening.com
washingtonfeed.com
washingtonpost.com.co
wattsupwiththat.com
wazanews.tk
wcpm3.com
webdaily.com
weconservative.com
weekendherald.com
weekendpoliticalnews.com
werk35.com

westernsentinel.com
westfieldpost.com
wetheproudpatriots.com
wftj8news.com
whale.to
whatdoesitmean.com
whatreallyhappened.com
whatsupic.com
whydontyoutrythis.com
winningdemocrats.com
witscience.org
wleb21.com
wm21news.com
wmacnews.com
wmb36.com
wnd.com
world-politicus.com
worldaffairsbrief.com
worldinformation24.info
worldnewscircle.com
worldnewsdailyreport.com
worldnewspolitics.com
worldpoliticsnow.com
worldpoliticus.com
worldtruth.tv
wrejnews.com
wrpm33.com
wrpt16.com
wtoe5news.com
wy21news.com
x22report.com
xbn-news.com
yesimright.com
yiannopoulos.net
yournewswire.com
zootfeed.com
